# Analogs of α‐conotoxin PnIC selectively inhibit α7β2‐ over α7‐only subtype nicotinic acetylcholine receptors via a novel allosteric mechanism

**DOI:** 10.1096/fj.202302079

**Published:** 2023-12-31

**Authors:** Andrew A. George, Sabin J. John, Linda M. Lucero, J. Brek Eaton, Ekta Jaiswal, Sean B. Christensen, Joanna Gajewiak, Maren Watkins, Yiwei Cao, Baldomero M. Olivera, Wonpil Im, J. Michael McIntosh, Paul Whiteaker

**Affiliations:** ^1^ Department of Pharmacology and Toxicology, School of Medicine Virginia Commonwealth University Richmond Virginia USA; ^2^ Department of Life Sciences University of Bath Bath UK; ^3^ Department of Neurobiology Barrow Neurological Institute Phoenix Arizona USA; ^4^ School of Biological Sciences University of Utah Salt Lake City Utah USA; ^5^ Department of Chemistry Lehigh University Bethlehem Pennsylvania USA; ^6^ George E. Wahlen Veterans Affairs Medical Center Salt Lake City Utah USA; ^7^ Department of Psychiatry University of Utah Salt Lake City Utah USA

**Keywords:** allosteric, Alzheimer's disease, basal forebrain cholinergic neuron, *Conus*, nicotinic

## Abstract

This study was undertaken to identify and characterize the first ligands capable of selectively identifying nicotinic acetylcholine receptors containing α7 and β2 subunits (α7β2‐nAChR subtype). Basal forebrain cholinergic neurons express α7β2‐nAChR. Here, they appear to mediate neuronal dysfunction induced by the elevated levels of oligomeric amyloid‐β associated with early Alzheimer's disease. Additional work indicates that α7β2‐nAChR are expressed across several further critically important cholinergic and GABAergic neuronal circuits within the central nervous system. Further studies, however, are significantly hindered by the inability of currently available ligands to distinguish heteromeric α7β2‐nAChR from the closely related and more widespread homomeric α7‐only‐nAChR subtype. Functional screening using two‐electrode voltage‐clamp electrophysiology identified a family of α7β2‐nAChR‐selective analogs of α‐conotoxin PnIC (α‐CtxPnIC). A combined electrophysiology, functional kinetics, site‐directed mutagenesis, and molecular dynamics approach was used to further characterize the α7β2‐nAChR selectivity and site of action of these α‐CtxPnIC analogs. We determined that α7β2‐nAChR selectivity of α‐CtxPnIC analogs arises from interactions at a site distinct from the orthosteric agonist‐binding site shared between α7β2‐ and α7‐only‐nAChR. As numerous previously identified α‐Ctx ligands are competitive antagonists of orthosteric agonist‐binding sites, this study profoundly expands the scope of use of α‐Ctx ligands (which have already provided important nAChR research and translational breakthroughs). More immediately, analogs of α‐CtxPnIC promise to enable, for the first time, both comprehensive mapping of the distribution of α7β2‐nAChR and detailed investigations of their physiological roles.

## Graphical abstract

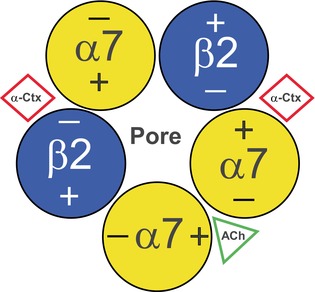

Analogs of α‐conotoxin PnIC selectively inhibit α7β2‐ over α7‐only subtype nicotinic acetylcholine receptors via a novel allosteric mechanism.Andrew A. George, Sabin J. John, Linda M. Lucero, J. Brek Eaton, Ekta Jaiswal, Sean B. Christensen, Joanna Gajewiak, Maren Watkins, Yiwei Cao, Baldomero M. Olivera, Wonpil Im, J. Michael McIntosh, & Paul Whiteaker*We describe α‐conotoxin PnIC (α‐CtxPnIC) analogs with selectivity towards nicotinic acetylcholine receptors containing α7 and β2 subunits (α7β2‐nAChR). Until now almost impossible to distinguish from widely‐distributed α7‐only‐nAChR, α7β2‐nAChR appear to play important physiological roles in multiple neuronal populations. Unlike previously‐described α‐Ctxs that compete for agonist binding sites, α‐CtxPnIC analogs gain α7β2‐nAChR selectivity via a novel allosteric mechanism (illustrated above). This finding fundamentally expands the scope of α‐Ctx studies, and enables the first comprehensive studies of α7β2‐nAChR distribution and roles.

**Abbreviations**
α‐Ctx: α‐conotoxin
AChBP: acetylcholine‐binding protein
AD: Alzheimer's disease
BSA: bovine serum albumin
MD: molecular dynamics
nAChR: nicotinic acetylcholine receptor
oAβ: oligomeric amyloid‐β
SMD: steered molecular dynamics

## 1 INTRODUCTION

A complex family of nicotinic acetylcholine receptor (nAChR) subtypes is expressed in both central and peripheral locations, each subtype representing a pentameric complex of specific, homologous, protein subunits.^1^ The homology across subunits results in extensive overlap of functional and pharmacological profiles across nAChR subtypes, making them difficult to distinguish. Without the ability to positively and unequivocally identify an individual nAChR subtype, it is impossible to study conclusively its role(s) in normal and disease physiology. Serendipitously, venomous mollusks of the genus *Conus* have evolved short peptide scaffolds (“conotoxins,” used as venom components) with astonishing selectivity across closely related targets.^2^, ^3^ In particular, α‐conotoxins (α‐Ctxs) are highly selective antagonists across nAChR subtypes, binding competitively to the same subunit interfaces to which conventional agonists such as nicotine or acetylcholine (ACh) bind.^4^, ^5^, ^6^, ^7^ These competitive binding sites form between one face of an α subunit (by convention, referred to as the “principal” or “(+)” face) and the opposite (“complementary” or “(−)”) face of an adjacent subunit.^1^ Due to their exceptional selectivity across nAChR subtypes, α‐Ctxs have become established as invaluable pharmacological tools with promise for clinical translation.^5^, ^8^

However, the reliance of current α‐Ctx antagonists on a competitive mode of action limits their scope of application. One example is “accessory” subunits such as α5 that do not participate in competitive binding sites,^9^ but can markedly alter nAChR assembly and function,^10^, ^11^ with significant effects on human health.^12^, ^13^, ^14^, ^15^ Another example is the discovery that, as α7‐only‐ and α7β2‐nAChR subtypes (collectively, α7*‐nAChR) are activated only by agonist binding to α7(+)/(−)α7 subunit interfaces that are shared between the two subtypes, competitive ligands cannot reliably distinguish between them.^16^, ^17^, ^18^ This latter issue has taken on increasing significance in light of the accumulating evidence that interactions between α7*‐nAChR and toxic, oligomeric, forms of amyloid‐β_1‐42_ (oAβ_42_) contribute to memory changes early in Alzheimer's disease and perhaps to cholinergic influences on cognition more broadly.^19^ Critically, findings suggest that α7β2‐nAChR are expressed in basal forebrain cholinergic neurons of both mice and humans,^17^ and that oAβ_42_ interacts differentially with α7‐only and α7β2‐nAChR (with α7β2‐nAChR being perhaps a more important mediator of oAβ_42_'s deleterious effects^19^, ^20^). There is also evidence of potential expression of α7β2‐nAChR in other cholinergic neuron populations within the mesopontine tegmentum, nucleus accumbens, striatum, and olfactory tubercle, as well as in septo‐hippocampal GABAergic neurons that project to the hippocampus and GABAergic neurons of the hippocampus itself.^21^, ^22^, ^23^ Together, these studies show that α7β2‐nAChR are positioned to play important roles in normal physiology and disease, and that there is a pressing need for a method to definitively distinguish α7β2‐nAChR from α7‐only‐nAChR.

This study was inspired by discoveries that (1) features of competitive agonist‐binding interfaces are conserved at subunit interfaces that do not support nAChR activation by standard agonists, and (2) binding of ligands at these “noncanonical” interfaces can profoundly alter nAChR function. For example, we and others have shown that different assembly ratios of the same subunits can have very different pharmacological profiles.^24^, ^25^, ^26^ These stoichiometry differences are also physiologically significant: epilepsy‐associated α4 and β2 subunit mutants consistently alter the balance of (α4)_2_(β2)_3_‐ versus (α4)_3_(β2)_2_‐nAChR stoichiometry,^27^ and agonists capable of preferentially stimulating (α4)_3_(β2)_2_‐nAChR produce distinctive physiological effects.^28^ In another example, we have shown that the prototoxin lynx1 can functionally distinguish between nAChR subtypes that all share competitive agonist‐binding sites at α3/β4 subunit interfaces, by interacting differentially at other non‐agonist‐binding subunit interface sites.^29^ Accordingly, we hypothesized that it should be possible for α‐Ctx ligands to exert functional effects by binding to conserved features of non‐agonist‐binding nAChR subunit interfaces.

Given the urgent need to distinguish between α7‐only‐ and α7β2‐nAChR, we chose this pair of subtypes as the venue to test our hypothesis. We report the discovery of a family of α‐CtxPnIC analogs that are able to distinguish between the closely related α7‐only‐ and α7β2‐nAChR subtypes. Our data provide converging lines of evidence that α7β2‐nAChR‐selective α‐CtxPnIC analogs exhibit preferential binding to α7(+)/(−)β2 interfaces (i.e., have a noncompetitive/allosteric antagonist effect).

## 2 MATERIALS AND METHODS

All standard reagents were sourced from Sigma‐Aldrich, Inc. (St. Louis, MO, USA) unless specified otherwise.

### 2.1 Conopeptide synthesis

α‐Ctxs with a predicted two disulfide‐bridge structure were identified by gene cloning using previously described methods.^30^, ^31^, ^32^ A library of these conopeptides and their analogs was synthesized using solid‐phase Fmoc (9‐fluorenylmethyloxycarbonyl) protocols, and folded into the typical Cys1‐Cys3; Cys2‐Cys4 disulfide‐bridged configuration by using a two‐step oxidation protocol to direct disulfide bond formation.^33^ Correctly folded peptides were purified by reverse phase high‐performance liquid chromatography, and peptide identities were confirmed using matrix‐assisted laser desorption/ionization mass spectrometry.^31^ Validated α‐Ctxs were lyophilized and stored at −20°C until reconstituted for use.

### 2.2 Expression of human nAChR subtypes in *Xenopus laevis* oocytes

For the following individual human nAChR subunits (α3, α4, α6/3, α7, α9, α10, β2, β3, and β4), cDNA clones were codon optimized for vertebrate expression and synthesized (GeneArt, a division of Thermo Fisher Scientific, Waltham, MA, USA). Here, α6/3 refers to a chimeric subunit encoding a human α6‐nAChR N‐terminal (i.e., competitive agonist‐binding) domain fused to the remaining sequence of a human α3‐nAChR subunit. This approach retains the competitive agonist‐binding pharmacology of the α6 subunit while greatly improving functional expression in *X. laevis* oocytes.^34^ In addition, a concatenated human‐subunit α7β2‐nAChR construct was used to enforce the expression and correct co‐assembly of nAChR with the (α7)_3_(β2)_2_ stoichiometry and subunit order 5′‐α7‐β2‐α7‐β2‐α7‐3′. Subunits were connected using alanine–glycine‐serine repeats engineered to produce linker lengths of 40 ± 2 amino acids (including the C‐terminal tail of the adjacent subunit). At the cDNA level, linkers encoded unique restriction sites that allowed substitution of individual subunits. The full details of the production and composition of this concatenated construct are provided in Moretti et al.^17^ In addition, a sequence‐optimized human clone for the α7‐nAChR chaperone protein NACHO^35^ was also synthesized (Geneart). Sequences and identities of all nAChR constructs, as well as of NACHO, were confirmed by DNA sequencing and restriction digest analysis. Verified cDNA constructs were subcloned into the following expression vectors: α3, α6/3, β2, β3, and β4, pSGEMHE^36^; α7, α7β2, α4, pSHE (a version of pSGEMHE modified to add further restriction enzyme recognition sites to the multiple cloning site^37^); α9, α10, NACHO, pSGEM‐AMV (a variant of pSGEM that incorporates the 5′‐untranslated region of the Alfalfa mosaic virus, shown to enhance expression of α9α10‐nAChR^38^). Transcription of cRNAs encoding individual or concatenated subunits was performed using the mMessage mMachine T7 kit (Thermo Fisher Scientific), and the resulting cRNAs were isolated using the Qiagen RNeasy Clean‐Up kit (Qiagen, Valencia, CA). Further experimental details are provided in Moretti et al.^17^ Purity of the isolated cRNAs was checked using electrophoresis on a 1% agarose gel, with confirmed samples being stored at −80°C before use.

*X. laevis* oocytes were purchased from Ecocyte Bioscience (Austin, TX, USA) and, upon receipt, were incubated at 16°C. Microelectrodes (resistance = 2–6 MΩ) were pulled from glass capillaries and used as micropipettes to inject cRNA (injection volume = 46 nL). Defined nAChR subtypes were expressed by injecting cRNAs as follows: α3β2, α3β4, or α4β2 (1 ng each subunit cRNA in each case), α6/3β2β3 (5:5:2.5 ng of the respective subunit cRNAs), α7‐only (1 ng α7 subunit cRNA), α7β2 (20 ng concatemer and 1 ng NACHO cRNA), and α9α10 (9:1 ng of the respective subunit cRNAs). For oocytes expressing nAChR subtypes containing unlinked subunits, recordings were performed at least 3 days (α3β4‐ or α4β2‐nAChR), or at least 5 days (α3β2‐, α6/3β2β3‐, or α9α10‐nAChR) following mRNA injection. For oocytes expressing the concatenated α7β2‐nAChR, recordings were collected at a minimum of 6 days following injection.

### 2.3 Initial functional screening of α‐Ctx pools at α7‐only‐ and α7β2‐nAChR expressed in *X. laevis* oocytes

In order to rapidly identify α‐Ctxs with selectivity for α7β2‐ over α7‐nAChR, the α‐Ctx library was divided into pools. Each pool contained either eight or nine peptides, with each individual peptide present at a concentration of 100 nM (4× pools of nine peptides each +53x pools of eight peptides each). The 100 nM concentration was chosen as peptides that did not have a pronounced functional effect at this concentration would have too low potency to be of practical use. Pools were prepared in assay buffer: OR2 solution (concentrations in mM: NaCl 92.5, KCl 2.5, MgCl_2_·6H_2_O 1, CaCl_2_·2H_2_O 1, HEPES 5, pH 7.5), supplemented with 0.1% w/v bovine serum albumin (BSA; to reduce adsorption of the peptides to the apparatus) and 1.5 μM atropine (to block any potential muscarinic responses). Two‐electrode voltage‐clamp (TEVC) recordings were used to assess the antagonist efficacy of each pool against ACh‐stimulated function of α7‐only‐ or α7β2‐nAChR, expressed in *X. laevis* oocytes. Solution application (flow rate 300 mL/h) and switching were performed using a 16‐channel, gravity fed, perfusion system with automated valve control (AutoMate Scientific, Berkeley, CA, USA). Glass microelectrodes (resistances 3–9 MΩ) were filled with an internal solution of 3 mM KCl and used to impale oocytes for recording. Recordings were performed at room temperature (22°C), and oocytes were voltage‐clamped at −70 mV. Data were collected with Clampex v10.7 (Molecular Devices, San Jose, CA, USA); sampled at 10 kHz (low‐pass Bessel filter, 40 Hz; high‐pass filter, DC). Oocytes maintaining a sustained inward leak current of >100 nA under these conditions were discarded.

Experiments proceeded in two phases. First, stable nAChR function was established by sequential stimulation with 5–6 test applications of ACh (300 μM in assay buffer, 3 s duration, applied every 60 s). Between ACh applications, oocytes were washed with assay buffer alone. The final response at the end of the first phase was used as a baseline control for each oocyte. Second, once stable function was observed for each oocyte, the antagonist effect of applying an α‐Ctx pool was determined. Oocytes were exposed to a chosen peptide pool for 5 min, then stimulated one more time with 1 mM ACh. Antagonist efficacy was determined, for each individual oocyte, by normalizing peak ACh‐induced function after peptide application to baseline function.

For the peptide pools with the greatest antagonist efficacy against ACh‐induced function of α7*‐nAChR, the identities of the individual active peptides within each pool were determined. The same screening protocol was repeated, with the exception that each constituent α‐Ctx peptide within the pools of interest was applied individually at 100 nM.

### 2.4 Kinetic modeling of antagonist activity of α‐CtxPnIC variants at α7‐only‐ and α7β2‐nAChR expressed in *X. laevis* oocytes

Peptides that appeared in the initial screen to have antagonist selectivity for α7β2‐ over α7‐only‐nAChR were selected for further testing. As noted in our earlier publication,^39^ the presence of multiple antagonist‐binding sites per receptor will result in a nonlinear relationship between antagonist occupancy of the receptor and the efficacy of the resulting block. Performing a kinetics analysis of antagonist activity can provide valuable insights into the number of antagonist sites present per receptor.^39^ All kinetics analyses were performed using SigmaPlot V14.0.3.192 (Inpixion, Palo Alto, CA, USA).

Kinetics data were collected using a variant of the *X. laevis* TEVC approach described for functional screening (preceding section). As individual experiments lasted several hours, modifications were made to maximize oocyte viability and the stability of nAChR function throughout the duration of the recordings. These modifications were (1) vacuum de‐gassing of assay buffer (to prevent formation of bubbles within the recording apparatus that could disrupt function or adversely impact oocyte survival), (2) allowing oocytes to acclimate to room temperature before initiating recording (to reduce variation in function during the initial stabilization phase of each recording), (3) extending the stabilization phase (to ensure consistent functional stability of ACh‐induced nAChR currents), and (4) slowing the solution application rate to 200 mL/h (to conserve peptide during prolonged application periods). Kinetics determinations were performed in three phases. First, stable nAChR function was established by sequential stimulation with test applications of ACh (1 mM in assay buffer, 3 s duration, applied every 60 s). Between ACh applications, oocytes were washed with assay buffer alone. In a modification of the functional screening approach, however, this stabilization phase was extended to a minimum of 60 min before the peptide of interest was applied. The mean of the last five responses at the end of this extended stabilization phase was used as a baseline control for each oocyte. In the second phase, onset of functional block by the α‐Ctx under consideration was observed. Oocytes were exposed to the peptide under observation for 57 s, then stimulated again with ACh (300 μM, 3 s). This cycle of peptide exposure and agonist stimulation was repeated until antagonist block approached a stable plateau. In the third phase, recovery from functional block was monitored. The same protocol was employed as just described (onset of block) except that, in this case, oocytes were washed with assay buffer between applications of ACh. Recovery was followed until at least 50% of baseline function was restored.

Kinetics analysis was performed in a similar manner to that detailed in Whiteaker et al.^39^ For analysis of off‐rate kinetics, recovery data were fit to:

(1) $$A_{t}=c+\%\text{Block}{\left(1-e^{-{\mathrm{tk}}_{\mathrm{off}}}\right)}^{N_{\mathrm{off}}}$$

where *A*_*t*_ is activity at time *t*, *c* is any remaining response in the presence of the peptide of interest at the time that washout begins, %*Block* is the percentage by which function was reduced at the point that washout is initiated (as a percentage of control function, defined before peptide was applied), *k*_*off*_ is the off‐rate constant, and *N*_*off*_ is the number of binding sites on the receptor from which the peptide must disassociate before function can recover. As noted in our previous work, the time‐course of recovery from peptide block will exhibit a lag when *N* > 1, and can provide an estimate of the value of *N*_*off*_.

For analysis of functional blockade two different equations were used, depending on whether onset of peptide‐induced block also exhibited a lag phase.

In cases where a lag phase of onset of block was observed, data were fit to:

(2) $$A_{t}=c+\%\text{Block}\left(1-{\left(1-e^{-{\mathrm{tk}}_{\mathrm{obs}}}\right)}^{N_{\mathrm{obs}}}\right)$$

where *k*_*obs*_ is the observed rate at which the applied peptide exerts its antagonist effect and %*Block* is, in this case, the percentage by which control function is predicted to be inhibited at equilibrium. Similar to Equation (1), the lag in onset can be used to determine the value of *N*_*obs*_ (the number of receptor‐binding sites required to be occupied to induce block of function).

In cases where onset of block was immediate (i.e., no lag phase was observed), data were fit to:

(3) $$A_{t}=c+\%\text{Block}{\left(e^{-{\mathrm{tk}}_{\mathrm{obs}}}\right)}^{N_{\mathrm{obs}}}$$

In this case, if binding of peptide to any one of multiple binding sites per receptor can induce functional antagonism, acceleration of functional block over what would be seen in a simple exponential decay will occur. This again allows a determination of the value of *N*_*obs*_ to be made (in this case, the number of receptor‐binding sites that can independently produce block of function).

The value of the true on rate (*k*_*on*_) differs from the observed decay constant *k*_*obs*_ since the latter is influenced by both the experimentally‐defined concentration of ligand ([L]) and *k*_*off*_:

(4) $$k_{\mathrm{obs}}=k_{\mathrm{on}}\left[L\right]+k_{\mathrm{off}}$$

Rearranging [4], the value of *k*_*on*_ may be calculated:

(5) $$k_{\mathrm{on}}=\frac{k_{\mathrm{obs}}-k_{\mathrm{off}}}{\left[L\right]}$$

The values of *k*_*off*_ and *k*_*on*_ were then used to determine the disassociation constant (*K*_*d*_) of each peptide:

(6) $$K_{d}=\frac{k_{\mathrm{off}}}{k_{\mathrm{on}}}$$

### 2.5 Molecular dynamics (MD) simulations, initial model building and identification of residues potentially responsible for differential peptide interactions at α7(+)/(−)α7 versus α7(+)/(−)β2 nAChR subunit interfaces

To build the model of (α7)_5_ homopentamer in complex with α‐CtxPnIC, we performed a search of nAChR in the Protein Data Bank (PDB), and selected the cryo‐electron microscopy (cryo‐EM) structure of pentameric α7‐only‐nAChR in complex with α‐bungarotoxin (PDBID:7KOO) as a template.^40^ We removed α‐bungarotoxin bound to the agonist‐binding site and truncated the transmembrane and cytoplasmic domains of nAChR to retain only the extracellular domain for our modeling and simulation study. There is no crystallographic or cryo‐EM structure of (α7)_3_(β2)_2_ heteropentamer, so we used the cryo‐EM structure of pentameric (α4)_3_(β2)_2_‐nAChR (PDBID:6CNK) to build a model of (α7)_3_(β2)_2_‐nAChR.^41^ We extracted the α7 subunit from the (α7)_5_ homopentamer, and performed structural alignment to identify the superposition of α7 subunit onto three α4 subunits in (α4)_3_(β2)_2_ heteropentamer. We combined the coordinates of three aligned α7 subunits with those of two existing β2 subunits to generate a (α7)_3_(β2)_2_ heteropentamer model.

To determine the binding pose of α‐CtxPnIC analogs in the agonist‐binding site, we searched nAChR‐conotoxin and acetylcholine‐binding protein (AChBP)‐conotoxin complex structures in the PDB. As there is no PDB structure with the exact same sequence as α‐CtxPnIC (GCCSHPPCFLNNPDYC), we selected the structure of AChBP in complex with a variant of α‐CtxPnIA (α‐CtxPnIA[A10L;D14K], which has the related sequence GCCSLPPCALNNPKYC; (PDBID:2BR8)) as a template.^42^ Two neighboring AChBP subunits constituting the α‐CtxPnIA[A10L;D14K] binding site were aligned onto the neighboring α7(+)/(−)α7 subunits in (α7)_5_ homopentamer and α7(+)/(−)β2 subunits in (α4)_3_(β2)_2_ heteropentamer, respectively, and the calculated rotation and translation matrices were used to obtain the new coordinates of α‐CtxPnIA[A10L;D14K] placed in the α7(+)/(−)α7 and α7(+)/(−)β2 interfaces.

Next, we replaced the side chains of residues that are different between α‐CtxPnIA[A10L;D14K] and α‐CtxPnIC to convert α‐CtxPnIA[A10L;D14K] to α‐CtxPnIC wild type (WT), α‐CtxPnIC[S4R], and α‐CtxPnIC[L10Y]. To confirm the binding pose inferred from the existing nAChR/AChBP‐conotoxin complex structures, we built alternative models with an upside down binding pose in which α‐CtxPnIC was flipped to swap the α‐carbon positions of L10 and S4. We generated multiple conformations of flipped α‐CtxPnIC with small differences between each other, and selected the ones with negligible bad contacts with nAChR to make the alternative models.

We carried out molecular dynamics (MD) simulations to investigate the motions of α‐CtxPnIC variants in the α7(+)/(−)α7 and α7(+)/(−)β2 interfaces and to identify key residue pairs contributing to binding interactions. The modeled complex structures of α‐CtxPnIC variants bound to (α7)_5_ and (α7)_3_(β2)_2_ were solvated in the TIP3P water model,^43^ and K^+^ and Cl^−^ ions at 0.15 M were added to neutralize the system. The van der Waals interactions were smoothly switched off at 10–12 Å by a force‐based switching function^44^ and the long‐range electrostatic interactions were calculated using the particle‐mesh Ewald method.^45^ Each system was first equilibrated in the canonical ensemble (NVT) for 125 ps. The production run was performed in the isothermal–isobaric ensemble (NPT) at 310.15 K and 1 bar with hydrogen mass repartitioning^46^ and 4 femtosecond time steps. Each of the six combinations (one of (α7)_5_ and (α7)_3_(β2)_2_ in complex with one of α‐CtxPnIC WT, [S4R] and [L10Y]) was simulated for 1 μs. The simulations were performed with OpenMM package^47^ and the CHARMM36(m) force field.^48^ The simulation systems and input files were generated by CHARMM‐GUI *Solution Builder*.^49^, ^50^ VMD was used for structure visualization,^51^ and MDTraj python library^52^ was used for the analysis of MD trajectories. The distance between each residue pair consisting of one from α‐CtxPnIC and the other from the α7(+)/(−)α7 or α7(+)/(−)β2 interface was calculated based on the closest heavy atoms, and the interacting residue pairs were identified by a distance cutoff of 4.5 Å. We calculated the frequencies of interacting residue pairs, and observed that the side chain of residue 10 (L10 in α‐CtxPnIC itself, or Y10 in α‐CtxPnIC[L10Y]), is oriented toward the inside of the binding site and continuously interacts with multiple residues located deep in the binding pocket. Several of these residues are non‐conserved: T105, L108 and Y117 on the α7(−) subunit, and their counterparts S108, V111 and W120 on the β2(−) subunit. Another two residues located at the entrance of the binding pocket, T76 and Q116 on the α7(−) subunit, and their counterparts K79 and F119 on the β2(−) subunit, are considered important as they also are non‐conserved, and are likely to be located on the pathway of ligand dissociation. These two sets of residues were further investigated (next section).

### 2.6 Kinetic modeling of antagonist activity of α7β2‐nAChR‐selective α‐CtxPnIC variants at α7β2‐nAChR containing mutant α7(+)/(−)β2 subunit interfaces

The initial MD simulation data (preceding section) indicated that two clusters of amino acid residues were both non‐conserved between α7(−) and β2(−) subunit faces, and were found in close proximity to the putative binding site of α‐CtxPnIC analogs (i.e., at the pair of α7(+)/(−)β2 interfaces located within the (α7)_3_(β2)_2_‐nAChR). Accordingly, we exchanged these non‐conserved β2 subunit (−)‐face residues for their equivalents in the α7 subunit (−)‐face, creating two β2 subunit variants:

$$\text{β2}\left[K7\text{9T},F119Q\right]\;\left({}^{“}\text{outer}\;{\text{pocket}}^{”}\right)$$

$$\text{β2}\left[S108T,V111L,W120Y\right]\;\left({}^{“}\text{inner}\;{\text{pocket}}^{”}\right)$$

Four new β2 subunit cDNAs were codon optimized for vertebrate expression and synthesized (GeneArt). The first two constructs encoded the “outer pocket” variant complete with peptide linkers containing unique restriction sites allowing insertion into the existing 5′‐α7‐β2‐α7‐β2‐α7‐3′ concatenated (α7)_3_(β2)_2_‐nAChR construct at either position 2 (5′ end encoded an XbaI site, 3′ end encoded an AgeI site) or position 4 (5′ end encoded an XhoI site, 3′ end encoded a NotI site). The second two constructs encoded the “inner pocket” variant accompanied by the same peptide linkers, which incorporated the same unique restriction sites. The new subunit variant constructs were used to produce two new (α7)_3_(β2)_2_‐nAChR variants. In the first (α7)_3_(β2)_2_‐nAChR variant, both the position 2 and position 4 β2 subunits were replaced with β2 subunits harboring the set of “outer pocket” amino acid substitutions. In the second (α7)_3_(β2)_2_‐nAChR variant, both the position 2 and position 4 β2 subunits were replaced with β2 subunits harboring the set of “inner pocket” amino acid substitutions. In each case, subunit replacement was achieved by restriction digestion of the parent (α7)_3_(β2)_2_‐nAChR construct using the corresponding unique restriction digestion sites, and ligation of the desired β2‐subunit variant into the intended position (an identical approach to that described in George et al.^29^).

As for the parent (α7)_3_(β2)_2_‐nAChR construct, subunit identity and correct positioning of subunits within the resulting cDNA constructs were confirmed by DNA sequencing and restriction digest analysis. Functional expression of these two new α7β2‐nAChR inner and outer pocket mutants was accomplished using precisely the same approach as detailed earlier for the unaltered α7β2‐nAChR concatenated construct (expression in *X. laevis* oocytes, following injection of 20 ng mRNA encoding the receptor variant of interest, along with 1 ng of NACHO mRNA).

In order to assess whether agonist activation was altered by the introduction of these amino acid substitutions at the β2 nAChR subunit (−) interfaces, ACh concentration‐response curves were collected using TEVC. Escalating doses of ACh in assay buffer (10 μM – 10 mM, in half‐log steps) were applied to oocytes expressing either the original unaltered α7β2‐nAChR construct, or one of the two new (α7)_3_(β2)_2_‐nAChR variants. Stimulations lasted 3 s, and were applied sequentially, 60 s apart. The data resulting from each individual oocyte were fitted to a standard logistic concentration‐response model (SigmaPlot V14.0.3.192), in order to obtain values of maximum agonist‐induced current (I_max_), ACh EC_50_, and hillslope (n_H_).

Next, for two new (α7)_3_(β2)_2_‐nAChR variants, and also for the original unaltered α7β2‐nAChR construct, kinetics of association and disassociation were assessed for the [S4R] and [L10Y] analogs of α‐CtxPnIC (i.e., those previously identified as having the greatest selectivity for α7β2‐ over α7‐only‐nAChR subtypes). An identical approach was taken in this case, as was detailed in the earlier “Kinetic modeling of antagonist activity of α‐CtxPnIC variants…” section.

### 2.7 Steered molecular dynamics (SMD) simulations, disassociation of α‐CtxPnIC[S4R] or α‐CtxPnIC[L10Y] from α7(+)/(−)α7‐, α7(+)/(−)β2‐, α7(+)/(−)β2[“inner pocket” variant]‐, or α7(+)/(−)β2[“outer pocket” variant]‐subunit interfaces

All‐atom SMD simulations were performed on all combinations of nAChR variants (i.e., α7(+)/(−)α7, α7(+)/(−)β2‐, α7(+)/(−)β2[“inner pocket”], and α7(+)/(−)β2[“outer pocket”]) in complex with α‐CtxPnIC variants (i.e., WT, [S4R], and [L10Y]). In addition to the overall simulation details mentioned earlier, the pulling forces were applied to the center of mass (COM) of nAChR pentamer and the COM of α‐CtxPnIC. In the pulling process, the spring constant was set to 5 kcal/mol/Å^2^ and the speed was 0.5 Å/ns to have the two COMs moving apart from each other. The SMD simulations terminated at 30 ns when α‐CtxPnIC completely detached from the binding interface. The simulation input files were generated by CHARMM‐GUI *Enhanced Sampler*.^53^ For each system, we first ran a conventional MD simulation for 500 ns, and ten snapshots sampled uniformly in the time series were then used to initialize ten independent SMD simulations for better statistics.

### 2.8 Functional screening of α‐CtxPnIC analogs at non‐α7*‐nAChR subtypes expressed in *X. laevis* oocytes

Further screening was performed to determine the selectivity of α‐CtxPnIC analogs across a range of non‐α7*‐nAChR subtypes (α3β2, α3β4, α4β2, α6/3β2β3, and α9α10). These were expressed in *X. laevis* oocytes as described earlier. Functional screening was performed using a similar approach to that described for the initial screen for activity at α7‐only‐ and α7β2‐nAChR.

First, stable nAChR function was established by sequential stimulation with test applications of ACh (3 s application time, 60 s between applications). For oocytes expressing α3β2‐, α3β4‐, α4β2‐ or α6/3β2β3‐nAChR, stable function was obtained within 5–6 stimulations (test pulses used ACh concentrations of 10 μM (α4β2‐ or α6/3β2β3‐nAChR), 30 μM (α3β2‐nAChR), or 1 mM (α3β4‐nAChR). These ACh concentrations were chosen to provide large functional responses from the subtypes of interest without inducing functional desensitization across multiple stimulations. For oocytes expressing α9α10‐nAChR, stable function was reached within 10 stimulations (1 mM ACh). In all cases, responses at the end of this initial stabilization phase provided baseline control function. Second, the α‐CtxPnIC analog of interest (300 nM) was applied for 5 min. At the end of the peptide application time, function induced by a further ACh test pulse was assessed. Antagonist efficacy was determined, for each individual oocyte, by normalizing peak ACh‐induced function after peptide application to baseline function.

### 2.9 Statistical analyses

Kinetics and functional activation parameters were compared across experimental groups using either one‐way or two‐way ANOVA (SigmaPlot V14.0.3.192).

## 3 RESULTS

### 3.1 Initial screening to identify peptide pools with high antagonist efficacy at α7*‐nAChR

**FIGURE 1:**
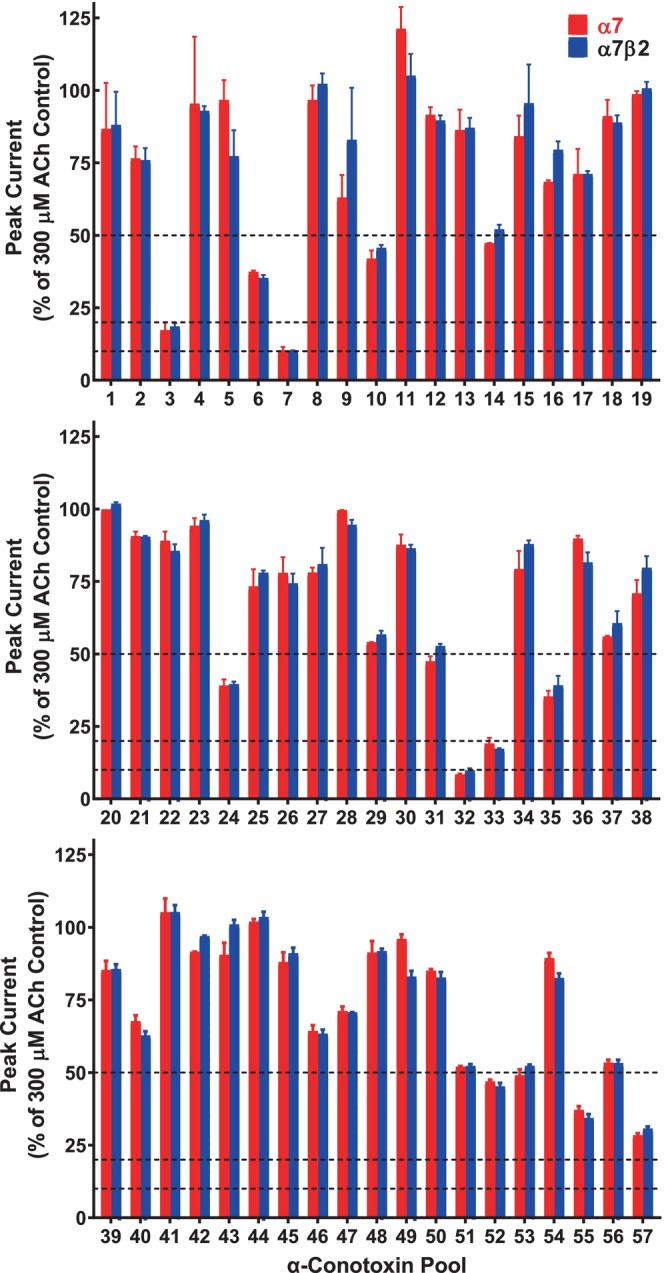
Initial screening of α‐Ctx pools for α7*‐nAChR activity: The peptide library was divided into 57 pools, each containing 8–9 peptides (at 100 nM concentration of each constituent peptide). Antagonist activity of each pool was determined at human α7‐only‐ or α7β2‐nAChR subtypes expressed in *X. laevis* oocytes, with function assessed using two‐electrode voltage‐clamp electrophysiology. Stable agonist‐induced control function was first established by repeated application of ACh (300 μM). Antagonist efficacy was determined for each pool by measuring the residual response to a further ACh (300 μM) stimulation, after application of the pool for 3 min. The four pools with the greatest antagonist efficacy (numbers 3, 7, 32, 33) were selected for further investigation. Data represent mean ± SEM of 2–4 individual determinations (using different oocytes for each determination).

Fifty‐seven pools of 8–9 individual peptides were applied to either human α7‐only‐ or α7β2‐nAChR heterologously expressed in *X. laevis* oocytes, as detailed in the Methods section. As shown in Figure 1, the majority of the pools had <50% efficacy in antagonizing ACh‐induced (1 mM) peak function of either subtype. As constituent peptides were present within pools at 100 nM, this suggested relatively low affinity of the peptides within such pools for either α7‐only‐ or α7β2‐nAChR. In contrast, four pools (numbers 3, 7, 32, and 33) showed exceptional antagonist efficacy (reducing ACh‐evoked function by ≈80%–90% when compared to control function prior to peptide application). These highly active pools were chosen for further study.

### 3.2 Screening to identify individual peptides with potential selectivity between α7‐only‐ and α7β2‐nAChR

**FIGURE 2:**
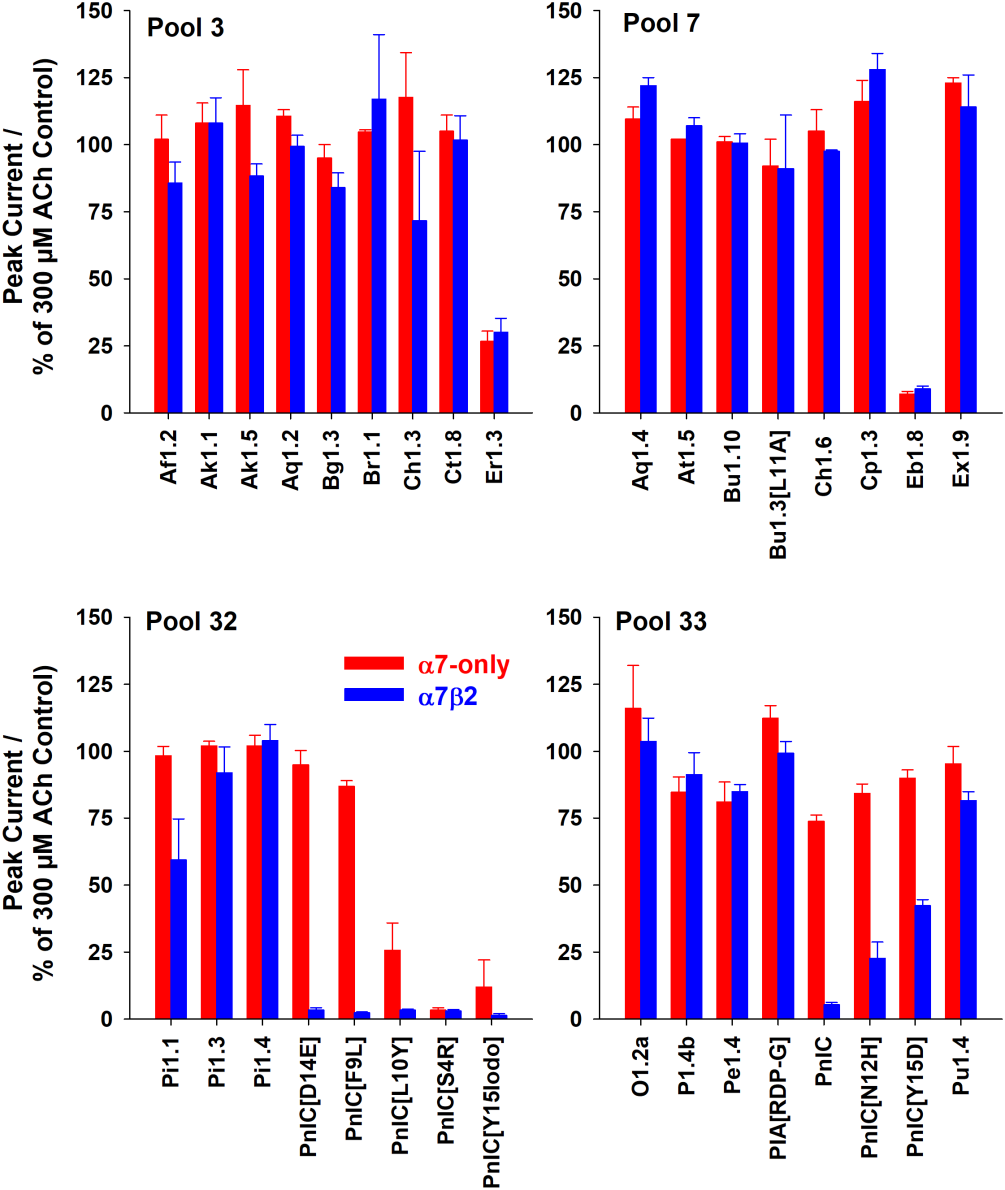
Identification of individual active peptides within the α‐Ctx pools with greatest α7*‐nAChR activity: The antagonist activity of the 33 individual peptides comprising the four most active pools identified in the initial screen (Figure 1) was determined at human α7‐only‐ or α7β2‐nAChR subtypes. The same screening protocol was employed except that, in this case, each α‐Ctx peptide of interest was applied individually at 100 nM. Data represent mean ± SEM of 2–4 discrete measurements (with separate oocytes used for each determination).

Having identified four pools of peptides that exhibited high potency for inhibiting function of α7*‐nAChR subtypes, we next sought to identify which individual peptide (or peptides) was/were responsible for the overall activity of each pool. Accordingly, we rescreened each of the individual peptides within each high‐potency pool at α7‐only‐ and α7β2‐nAChR. Individual peptides were applied at 100 nM (the same concentration that they were present within their respective pools). The outcomes are summarized in Figure 2. Antagonist activity of Pool 3 appeared to be produced by only one of the constituent peptides (α‐CtxEr1.3), which suppressed function of both α7‐only‐ and α7β2‐nAChR by ≈75% compared to control (similar to the activity of the whole pool; see Figure 1). A similar outcome was observed for Pool 7, where a single peptide (α‐CtxEb1.8) reduced function of both α7*‐nAChR subtypes by ≈85% compared to control (again reproducing the effect of the entire pool; Figure 1). However, the findings for Pools 32 and 33 were very different. Each of these two pools contained multiple peptides (α‐CtxPnIC and seven analogs with individual amino acid substitutions; listed in Table 1) that suppressed ACh‐evoked responses at α7*‐nAChR. Strikingly, α‐CtxPnIC itself, as well as multiple analogs, appeared to serve as more efficacious antagonists of α7β2‐nAChR than of α7‐only‐nAChR. This simple two‐stage approach (first identifying pools of peptides with desired properties, followed by screening activity of individual peptides within the active pools), therefore, provided a robust and effective method of identifying a family of lead peptides for further characterization.

**TABLE 1:** Amino acid sequences of α‐Ctx peptides identified as having potent α7*‐nAChR antagonist activity or used in molecular dynamics model building.

Peptide name	Amino acid sequence
PnIC	GCCSHPPCFLNNPDYC#
PnIC[S4R]	GCCRHPPCFLNNPDYC#
PnIC[F9L]	GCCSHPPCLLNNPDYC#
PnIC[L10Y]	GCCSHPPCFYNNPDYC#
PnIC[N12H]	GCCSHPPCFLNHPDYC#
PnIC[D14E]	GCCSHPPCFLNNPEYC#
PnIC[Y15D]	GCCSHPPCFLNNPDDC#
PnIC[Y15 **iodo**]	GCCSHPPCFLNNPD **Y** C#
Er1.3	NGCCSNPACILNNPNQC#
Eb1.8	GCCSHPACRVHYPHVCY#
PnIA[A10L;D14K]	GCCSLPPCALNNPKYC#

*Note*: α‐CtxPnIC and seven variants were identified by initial screening as being high‐potency antagonists of α7*‐nAChR, with likely selectivity toward α7β2‐ over α7‐only‐nAChR. The amino acid sequences of these eight α‐CtxPnIC variants are provided (letters in red highlight amino acid substitutions in the analogs compared to the parent peptide). Also noted are amino acid sequences of two further peptides identified during the screen as being potent α7*‐nAChR antagonists but lacking selectivity between α7β2‐ and α7‐only‐nAChR (α‐CtxEr1.3 and α‐CtxEb1.8). The known structure of α‐CtxPnIA[A10L;D14K] was used in building molecular dynamics models since its sequence closely resembles that of α‐CtxPnIC. The amino acid sequence of α‐CtxPnIA[A10L;D14K] is also listed (letters underlined denote sequence differences from α‐CtxPnIC). # denotes an amidated C‐terminus.

### 3.3 Kinetics of antagonism of α‐CtxPnIC analogs at α7‐only‐ and α7β2‐nAChR

Prior studies have shown that kinetics of α‐Ctx antagonism can differ widely even between closely related nAChR subtypes. These differences in kinetics can enable determination of binding mechanisms and enhance the capacity of peptides to discriminate among nAChR subtypes. One such example is α‐CtxBuIA, which kinetically distinguishes between β2*‐ and β4*‐nAChR subtypes. Specifically, α‐CtxBuIA kinetically distinguished between subtypes sharing a common α subunit, but differing in the identity of the β subunit providing the complementary face of the peptide‐binding site.^54^ In the initial screening studies, peptides were applied for a fixed period (3 min) before antagonist efficacy was measured. Accordingly, we undertook an analysis of the kinetics of the antagonist effect of α‐CtxPnIC and its analogs, at both α7‐only‐ and α7β2‐nAChR.

**FIGURE 3:**
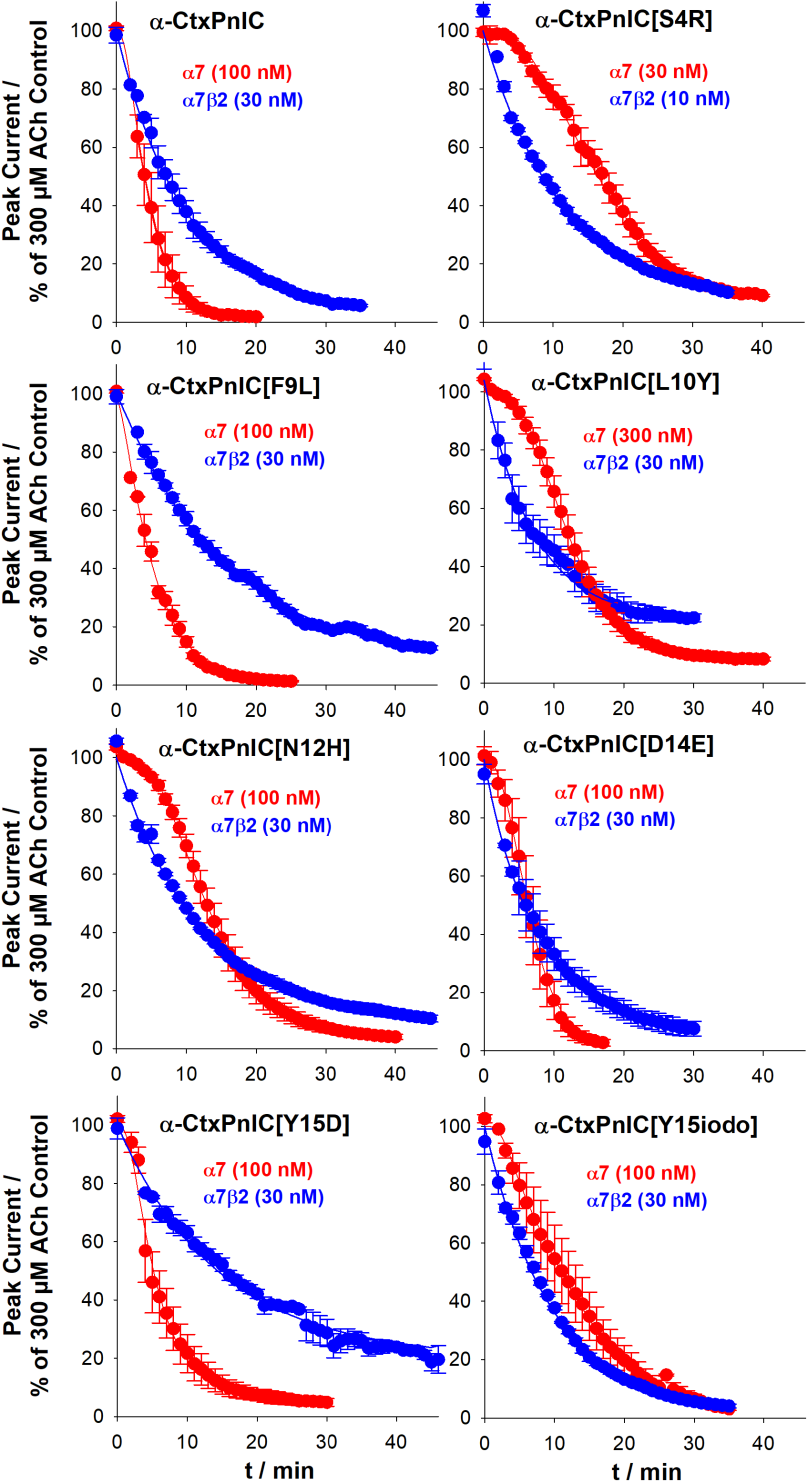
Delayed onset of antagonism by α‐CtxPnIC analogs at human α7‐only‐ compared to α7β2‐nAChR expressed in *X. laevis* oocytes: The time‐course of onset of antagonism of α7β2‐ or α7‐only‐nAChR function was observed for each of the α‐CtxPnIC analogs identified by screening as potentially selective toward α7β2‐ over α7‐only‐nAChR. Concentrations applied in each case are noted in each panel. nAChR function was measured using two‐electrode voltage‐clamp electrophysiology. Data were fit to multiple site kinetic models to assess the kinetic of onset of antagonism for each individual oocyte tested. The resulting values for observed association rate (*k*_*obs*_) and numbers of sites to which each analog had to associate in order to block function (*N*_*obs*_), along with statistical analyses, are summarized in Table 2. Note that onset of antagonism at α7‐only‐nAChR was characterized by a distinct lag phase for all peptides except for α‐CtxPnIC[F9L]. Best fit lines are shown in each panel, derived from the mean values of the parameters determined using curve fitting. Data points represent the mean ± SEM of 3–6 individual determinations, with each determination obtained from an individual oocyte, across a minimum of at least three separate batches of oocytes.

As illustrated in Figure 3, onset of functional block was relatively slow for α‐CtxPnIC and its analogs, at both α7‐only‐ and α7β2‐nAChR subtypes; most analogs required 20–30 min for antagonist efficacy to approach a plateau of activity. However, the most striking finding was that onset of block at α7‐only‐nAChR displayed a notable lag phase for all but one of the peptides (α‐CtxPnIC[F9L]). The presence of this lag phase is especially easy to see in the cases of the [S4R], [L10Y], and [N12H] analogs, where only a very gradual onset functional block is observed for at least 5 min following application of the peptide, followed by a faster phase of onset. As detailed in the Methods section, this lag is indicative of a requirement for more than one binding site to be engaged by the peptide antagonist in order to exert antagonism and can be used to determine the number of sites required to be occupied to block receptor function. Values of *k*_*obs*_ (observed rate of onset of block) and *N*_*obs*_ (number of sites required to block) arising from the kinetics analysis are summarized in Table 2. In contrast, no lag was seen in onset of block at α7β2‐nAChR for any of the peptides tested, or for the [F9L] analog at α7‐only‐nAChR. In fact, kinetics analysis indicated that onset of block was somewhat steeper in each case than would be expected for association of antagonist at a single site. As noted at in the Methods section, this is again indicative of multiple antagonist‐binding sites per receptor but, in the case of the α7β2‐nAChR, occupation of even a single site produced effective block of function. Values of *k*_*obs*_ and *N*_*obs*_ (in this case, number of sites per receptor that are available to produce block) calculated by the corresponding kinetics analysis are also summarized in Table 2. Overall, these results indicate that α‐CtxPnIC analogs can distinguish α7‐only‐ from α7β2‐nAChR on the basis of onset of antagonism. This effect is driven by the number of sites to which the peptides have to bind in order to block function.

**TABLE 2:** Kinetic analyses of α‐CtxPnIC and analog activity at α7β2‐ or α7‐only‐nAChR.

Peptide	*k*_*off*_/min^−1^ (×10^−3^)		*N*_*off*_		*k*_*obs*_/min^−1^ (×10^−3^)		*N*_*obs*_		*k*_*on*_/min^−1^ nM^−1^ (×10^−3^)		*K*_*d*_/nM		α7β2 Selectivity
	α7β2	α7‐only	α7β2	α7‐only	α7β2	α7‐only	α7β2	α7‐only	α7β2	α7‐only	α7β2	α7‐only	
α‐CtxPn1.2	7.15 ± 1.12	7.34 ± 0.91	1.66 ± 0.31	1.58 ± 0.25	75.6 ± 10.1	419 ± 73	1.39 ± 0.03	3.24 ± 0.35	2.23 ± 0.33	4.12 ± 0.74	3.3 ± 0.6	1.9 ± 0.4	*ns*
[S4R]	5.60 ± 0.33	**35.8** ± **6.5** ^‡‡‡^	1.12 ± 0.05	1.29 ± 0.29	68.3 ± 3.5	111 ± 10	1.31 ± 0.03	4.68 ± 0.92	6.74 ± 0.66	3.40 ± 1.04	0.83 ± 0.05	**15** ± 6***	18x
[F9L]	17.5 ± 2.1	**38.2** ± **3.4** ^‡‡^	1.55 ± 0.10	2.31 ± 0.22	54.4 ± 10.6	235 ± 10	1.32 ± 0.10	1.47 ± 0.18	1.23 ± 0.29	1.97 ± 0.13	15 ± 2	20 ± 3	*ns*
[L10Y]	27.7 ± 4.6	**264** ± **20** ^‡‡‡^	1.23 ± 0.21	1.92 ± 0.22	121 ± 433	186 ± 13	1.04 ± 0.17	4.58 ± 1.25	3.01 ± 1.05	**0.40** ± **0.14** ^†^	12 ± 3	**671** ± 136***	57x
[N12H]	18.5 ± 1.7	**66.1** ± **8.9** ^‡‡‡^	1.64 ± 0.05	2.41 ± 0.36	62.6 ± 0.8	176 ± 22.3	1.36 ± 0.02	5.97 ± 0.66	1.47 ± 0.05	1.10 ± 0.30	13 ± 2	**81** ± 19***	6.2x
[D14E]	3.60 ± 0.41	**6.67** ± **1.46** ^‡^	1.21 ± 0.05	1.21 ± 0.05	92.7 ± 20.0	351 ± 27.9	1.53 ± 0.06	5.80 ± 1.35	2.97 ± 0.67	3.45 ± 0.29	1.4 ± 0.3	2.0 ± 0.6	*ns*
[Y15D]	17.9 ± 2.4	**67** ± **14** ^‡‡‡^	1.05 ± 0.10	2.05 ± 0.19	47.5 ± 7.0	396 ± 80.9	1.49 ± 0.13	4.33 ± 0.73	2.60 ± 0.75	3.29 ± 0.84	9.9 ± 2.1	**27** ± 3*	2.7x
[Y15iodo]	3.43 ± 0.68	**10.6** ± **0.7** ^‡‡^	1.58 ± 0.11	2.16 ± 0.46	71.5 ± 2.9	246 ± 99	1.51 ± 0.06	5.78 ± 0.99	2.27 ± 0.11	1.65 ± 1.00	1.5 ± 0.3	**6.5** ± 2.6*	4.3x

*Note*: For each α‐CtxPnIC analog, multiple site models were used to assess the kinetics of recovery from, and onset of, antagonism of α7β2‐ or α7‐only‐nAChR (see Methods, and Figures 3 and 4). The values resulting from the recovery model of the true disassociation rate (*k*_*off*_) and numbers of sites from which peptides had to disassociate in order for function to recover (*N*_*off*_) are listed, for each peptide at each subtype. Similarly, values of observed association rate (*k*_*obs*_) and numbers of sites to which each analog had to associate in order to block function (*N*_*obs*_) are provided for each peptide at each subtype (derived from the onset models). As described in the Methods section, *k*_*off*_ and *k*_*obs*_ were used to calculate the true association rate (*k*_*on*_), for each oocyte tested. Separate analyses, using two‐way ANOVA followed by *post hoc* analysis (Holm‐Sidak method) were used to investigate potential differences between α7β2‐ and α7‐only‐nAChR subtypes for each of the true association rates (*k*_*on*_; ^†^ *p* < .05) or disassociation rates (*k*_*off*_; ^‡^ *p* < .05, ^‡‡^ *p* < .01, ^‡‡‡^ *p* < .001), for each peptide. Disassociation constant (*K*_*d*_) values were calculated using *k*_*on*_ and *k*_*off*_ determined for each oocyte. The resulting *K*_*d*_ values were also analyzed using two‐way ANOVA followed by *post hoc* analysis (Holm‐Sidak method) to identify peptides with α7β2‐nAChR selectivity (*p < .05, **p < .01, ***p < .001, *ns* = no significant selectivity). All kinetic data are the mean ± SEM of 3–6 individual determinations, corresponding to individual oocytes. F‐values, degrees of freedom, etc. for the ANOVA analyses are summarized in the Results section.

**FIGURE 4:**
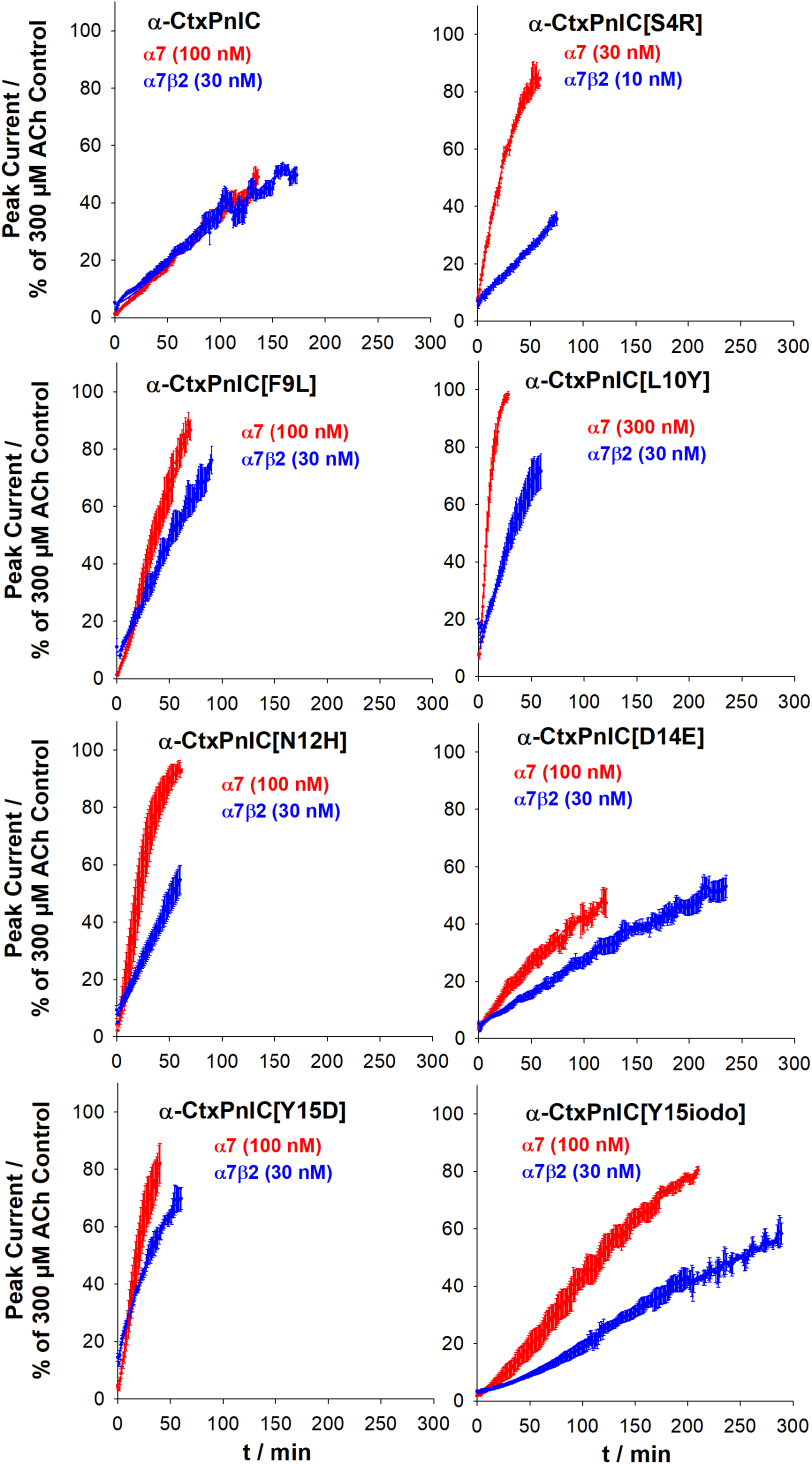
Slower recovery from antagonism by α‐CtxPnIC analogs at human α7β2‐ compared to α7‐only‐nAChR expressed in *X. laevis* oocytes: Kinetics of recovery from antagonism by each of the α‐CtxPnIC analogs were measured at α7β2‐ or α7‐only‐nAChR, using two‐electrode voltage‐clamp electrophysiology. Peptide concentrations are noted in each panel. Data were fit to a multiple site kinetic model to assess the kinetic of onset of antagonism for each individual oocyte tested. The resulting values for true disassociation rate (*k*_*off*_) and numbers of sites from which each analog had to disassociate in order to relieve block of function (*N*_*off*_), along with statistical analyses, are summarized in Table 2. Note that for most analogs, recovery of α7β2‐nAChR function was considerably slower than for α7‐only‐nAChR. Best fit lines are shown in each panel, derived from the mean values of the curve‐fitted parameters. Data points represent the mean ± SEM of 3–6 individual determinations, with each determination obtained from an individual oocyte, across a minimum of at least three separate batches of oocytes.

Recovery from block was also assessed, at both α7‐only‐ and α7β2‐nAChR subtypes, for α‐CtxPnIC and its analogs. As shown in Figure 4, rates of recovery were always slow for both subtypes, but varied widely between the peptides. Also notable is that recovery of function typically showed a lag phase (perhaps best illustrated in the case of the [Y15iodo] analog). This is indicative of a requirement for disassociation of more than one antagonist molecule before functional recovery can occur. Values of *k*_*off*_ (the true disassociation rate) and *N*_*off*_ (the number of sites per receptor from which antagonist must disassociate before function can be recovered) were derived as described in the Methods section, and are reported in Table 2. Two‐way ANOVA was used to consider the effect of nAChR subtype (α7‐only or α7β2) and peptide analog on *k*_*off*_. This analysis indicated that there was a statistically significant interaction between nAChR subtype and peptide analog on k_off_ (F [7, 43] = 69.63, *p* < .001). Considering main effects, both nAChR subtype (F [1, 43] = 162.31, *p* < .001) and analog (F [7, 43] = 6.23, *p* < .001) had significant effects on *k*_*off*_. Pairwise comparisons (Holm‐Sidak method) indicated that *k*_*off*_ differed significantly between α7‐only‐ and α7β2‐nAChR for every analog, with the sole exception of the parent peptide, α‐CtxPnIC itself (summarized in Table 2).

For each combination of peptide and nAChR subtypes, *k*_*off*_ values were used to calculate the corresponding *k*_*on*_ (true association rate) values (see Methods section for details; outcomes are reported in Table 2). In a similar manner to the preceding analysis, two‐way ANOVA was used to investigate the effect of nAChR subtype (α7‐only or α7β2) and peptide analog on *k*_*on*_. This revealed that there was a statistically significant interaction between nAChR subtype and peptide analog (F [7, 43] = 6.69, *p* < .001). Main effects analysis showed that nAChR subtype did not have a statistically significant effect on *k*_*on*_ (F [1, 43] = 2.66, *p* = .110), but that *k*_*on*_ was significantly affected by peptide analog (F [7, 43] = 8.61, *p* < .001). Pairwise *post‐hoc* comparisons (Holm‐Sidak method) indicated that *k*_*on*_ differed significantly between α7‐only‐ and α7β2‐nAChR for only one analog (α‐CtxPnIC[L10Y]; noted in Table 2).

Last, values of *k*_*off*_ and *k*_*on*_ were used to determine the disassociation constant (*K*_*d*_) of each peptide at each nAChR subtype (approach summarized in the Methods section, and the outcomes presented in Table 2). Paralleling the approach for *k*_*off*_ and *k*_*on*_, two‐way ANOVA was also used to analyze the effect of nAChR subtype (α7‐only‐ or α7β2) and peptide analog on *K*_*d*_. In this case, a statistically significant interaction was seen between analog and nAChR subtype (F [7, 43] = 12.26, *p* < .001). Both main effects of nAChR subtype and analog on *K*_*d*_ were seen (F [1, 43] = 89.46, *p* < .001, and F [7, 43] = 44.20, *p* < .001, respectively). Pairwise comparisons (Holm‐Sidak method) revealed that *K*_*d*_ differed significantly between α7‐only‐ and α7β2‐nAChR for five analogs ([S4R], [L10Y], [N12H], [Y15D], and [Y15iodo]). These outcomes are noted in Table 2, along with the degree of selectivity observed between α7‐only‐ and α7β2‐nAChR subtypes for each analog.

In summary, the kinetics analysis indicated that true association rates (*k*_*on*_) were quite similar across α‐CtxPnIC analogs, and between α7‐only‐ and α7β2‐nAChR, with the sole exception of α‐CtxPnIC[L10Y] (for which *k*_*on*_ was significantly faster at α7β2‐ than α7‐only‐nAChR). In strong contrast, disassociation rates (*k*_*off*_) varied widely across α‐CtxPnIC analogs, and between α7‐only‐ and α7β2‐nAChR (with *k*_*off*_ being significantly faster from α7‐only‐ than from α7β2‐nAChR for every analog tested, except α‐CtxPnIC itself). Accordingly, the higher binding affinity of multiple analogs at α7β2‐ over α7‐only‐nAChR (measured as the disassociation constant, *K*_*d*_) is primarily driven by their faster disassociation from (and consequently lower affinity for) α7‐only‐nAChR. This point is reinforced by the relatively narrow range of *K*_*d*_ values across all analogs for the α7β2‐nAChR subtype (1.4–15 nM = 10.7‐fold), compared to the range observed at the α7‐only subtype (1.9–671 nM = 353‐fold).

### 3.4 Modeling and simulation of nAChR‐α‐CtxPnIC complex and identification of the “inner pocket” and “outer pocket” residues for mutation experiments

**FIGURE 5:**
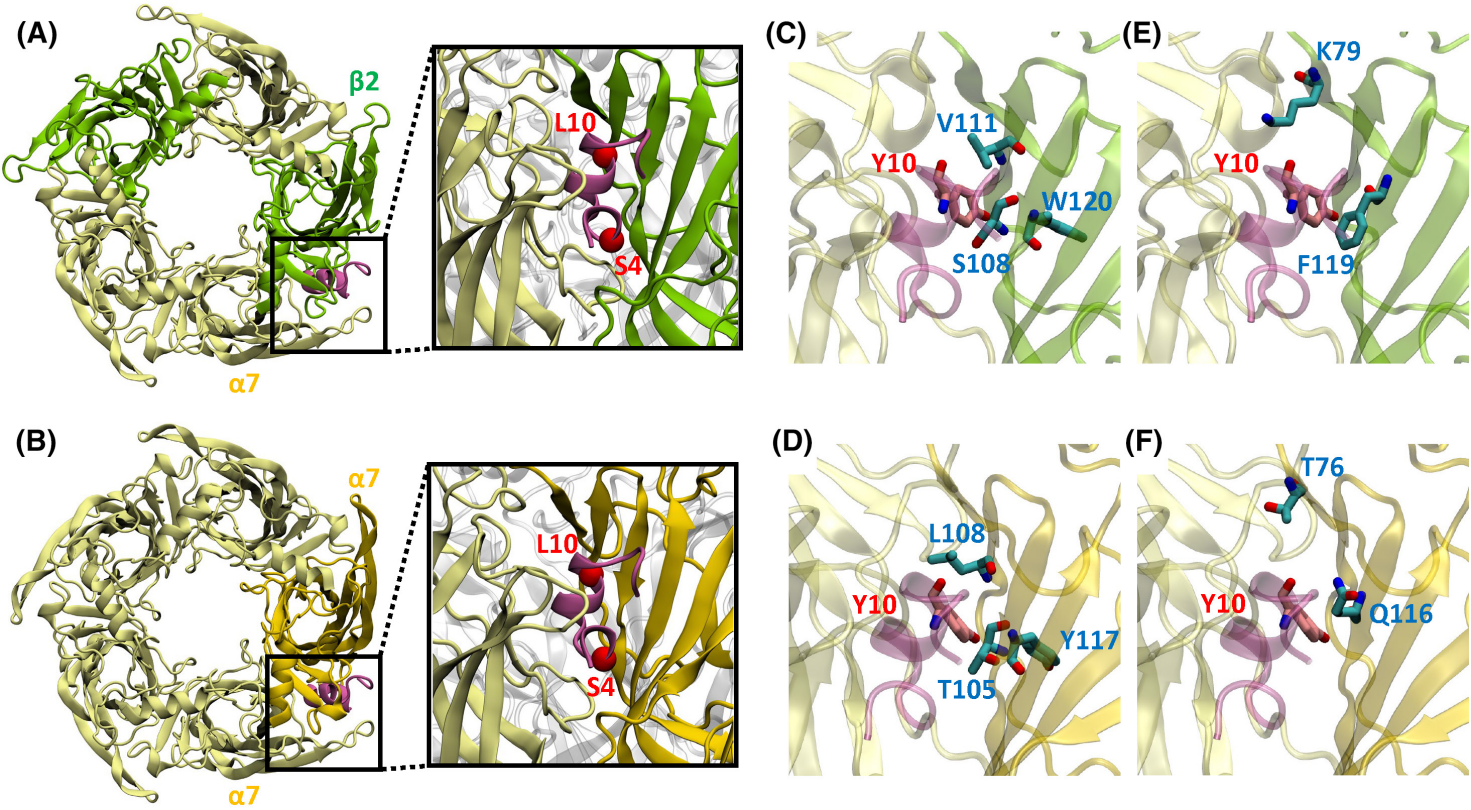
Modeling of (α7)_5_ and (α7)_3_(β2)_2_ pentamer, orientation of α‐CtxPnIC, and definition of “inner pocket” and “outer pocket” residues at putative α7(+)/(−)β2 binding site: (A) Heteromeric (α7)_3_(β2)_2_ in complex with α‐CtxPnIC. The α7 and β2 subunits are shown in pale yellow and green, respectively, and α‐CtxPnIC is shown in magenta. The S4 and L10 positions of α‐CtxPnIC are highlighted with red spheres. The orientation of α‐CtxPnIC is determined based on the PDB structures of AChBP in complex with the related α‐CtxPnIA[A10L;D14K]. (B) Homomeric (α7)_5_ in complex with α‐CtxPnIC. The α7 subunits are shown in pale yellow, with the α7(−) subunit at the binding site highlighted by golden yellow. (C, D) The “inner pocket” residues at the α7(+)/(−)β2 interface and the corresponding residues at the α7(+)/(−)α7 interface. Three “inner pocket” residues are shown in cyan, and Y10 of α‐CtxPnIC[L10Y] is shown in pink. (E, F) The “outer pocket” residues at the α7(+)/(−)β2 interface and the corresponding residues at the α7(+)/(−)α7 interface. Two “outer pocket” residues are shown in cyan.

To gain insight into the interactions between differing nAChR‐binding interfaces (i.e., α7(+)/(−)α7 or α7(+)/(−)β2) and α‐CtxPnIC analogs (WT, [S4R], or [L10Y]), we built a model structure of pentameric nAChR bound to one α‐CtxPnIC molecule for each combination (Figure 5A,B), and carried out MD simulation for 500 ns. Starting from an initial binding pose obtained based on a published PDB structure of α‐CtxPnIA[A10L;D14K] bound to AChBP (see methods and^42^), the WT α‐CtxPnIC, α‐CtxPnIC[S4R], and α‐CtxPnIC[L10Y] were all very stable in the binding site. The backbone root mean square deviation (RMSD) of α‐CtxPnIC analogs in all simulations was less than 3.5 Å. To confirm the binding pose of α‐CtxPnIC, we performed additional simulations starting from alternative models of nAChR‐α‐CtxPnIC complexes, built by flipping α‐CtxPnIC (detailed in Methods). α‐CtxPnIC in the resulting “upside down” pose was unstable and we observed large rotational and translational motions. The peptide either rotated back to the binding pose derived from PDB structures (Figure S1), or simply left the binding site within a short simulation time.

As the α7(+) subunits are the same as in the α7(+)/(−)α7 and α7(+)/(−)β2 interfaces, we focused on the complementary α7(−) and β2(−) subunits, and calculated the 2‐D contact map showing the interacting residue pairs consisting of one residue from α‐CtxPnIC and the other from the α7(−) or β2(−) subunit (Figure S2). Two residues were considered to be in contact if the minimum distance between their side‐chain heavy atoms was less than 4.5 Å. We observed that L10 in α‐CtxPnIC WT and [S4R] and Y10 in α‐CtxPnIC[L10Y] all continuously interacted with V111, F119 and L121 of β2(−) subunit, which correspond to L108, Q116 and L118 of α7(−) subunit. Y10 has a longer side chain that inserts deeper into the binding pocket. It interacts with W120 of β2(−) subunit but not with Y117 of α7(−) subunit, and it interacts with T105 of α7(−) subunit but not with S108 of β2(−) subunit. S108^β2(−)^, V111^β2(−)^, and W120^β2(−)^ are not conserved and their counterparts T105^α7(−)^, L108^α7(−)^, and Y117^α7(−)^ are buried deep in the binding site. Accordingly, they were grouped into one set, namely the “inner pocket” set (Figure 5C,D) and were selected for further investigation by site‐directed mutagenesis. In addition, F119^β2(−)^, L121^β2(−)^ and their equivalent residues Q116^α7(−)^ and L118^α7(−)^ are located near the entrance of the binding site. As L121^β2(−)^ and L118^α7(−)^ are the same amino acid, only F119^β2(−)^ and Q116^α7(−)^ could be further investigated in a site‐directed mutagenesis experiment. However, we noticed that K79^β2(−)^ and its counterpart T76^α7(−)^ are also located near the entrance and potentially could affect the association and dissociation of α‐CtxPnIC (together with the F119^β2(−)^/Q116^α7(−)^ pair). The mutation from Lys to Thr leads to a significant difference in both size and charge. Therefore, K79^β2(−)^, F119^β2(−)^, T76^α7(−)^, and Q116^α7(−)^ were grouped into the “outer pocket” set (Figure 5E,F) for further study in the next section.

### 3.5 Kinetic modeling of antagonist α‐CtxPnIC[S4R] and [L10Y] analogs at α7β2‐nAChR containing mutant α7(+)/(−)β2 subunit interfaces

As noted in the preceding section, two sets of amino acid residues were indicated to be (1) non‐conserved between the complementary (−) faces of α7 and β2 nAChR subunits, and (2) in close proximity to a putative binding site for α‐CtxPnIC analogs at the pair of α7(+)/(−)β2 subunit interfaces found only in the α7β2‐nAChR subtype. Accordingly, it seemed probable that one or both of these sets of non‐conserved amino acids could be important mediators of α‐CtxPnIC analog selectivity for α7β2‐ over α7‐only‐nAChR. To examine this possibility, we exchanged one or the other of these non‐conserved sets of amino acids at the β2(−) subunit face for their counterparts in the α7(−) subunit face, at both α7(+)/(−)β2 subunit interfaces. This produced two new constructs:

1. Outer pocket mutant α7β2‐nAChR (β2[K79T,F119Q] at both α7(+)/(−)β2 subunit interfaces)
2. Inner pocket mutant α7β2‐nAChR (β2[S108T, V111L,W120Y] at both α7(+)/(−)β2 subunit interfaces)

**FIGURE 6:**
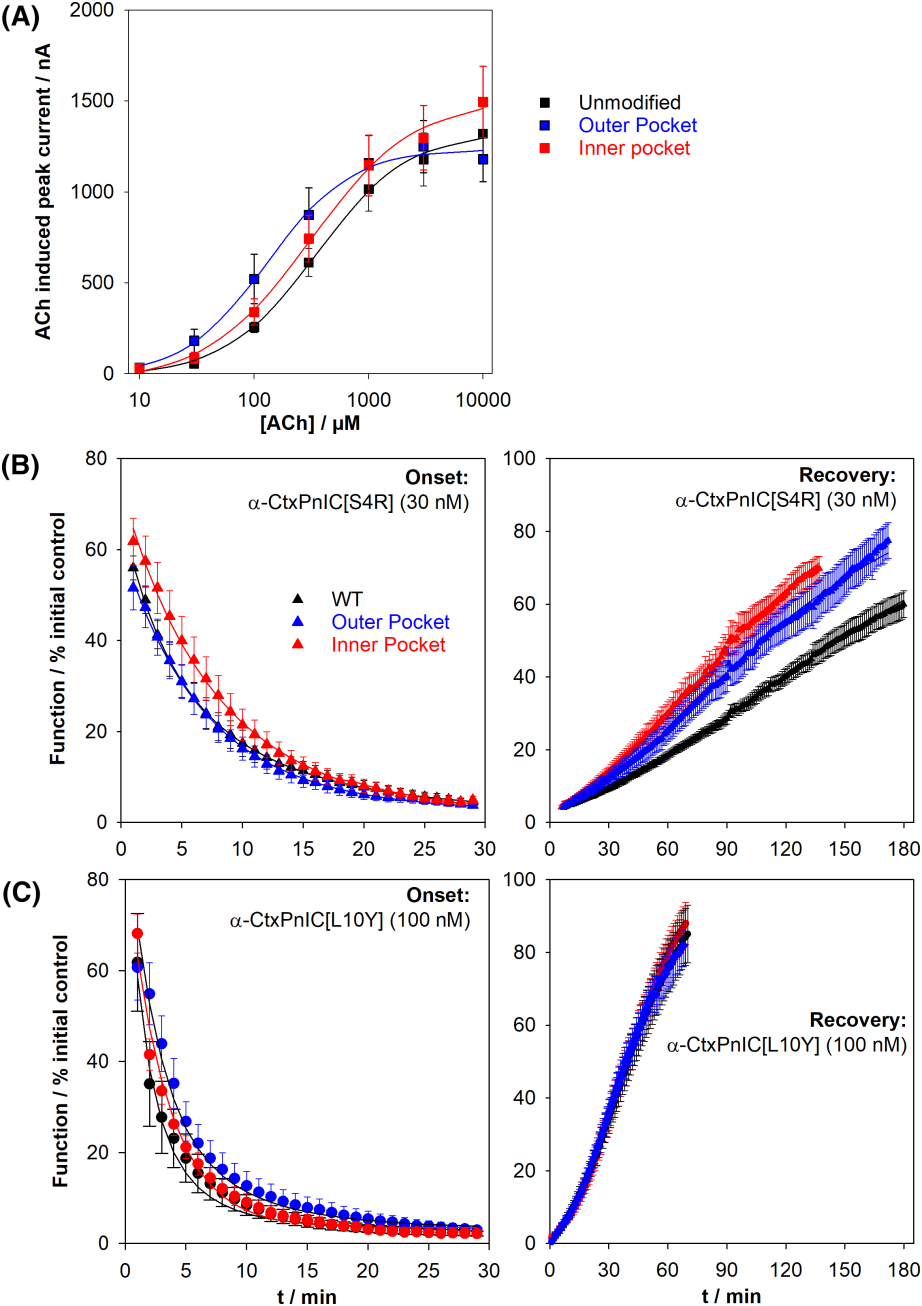
Functional characterization of “inner pocket” and “outer pocket” α7β2‐nAChR mutants expressed in *X. laevis* oocytes: Variants of human concatemeric α7β2‐nAChR were engineered to determine the functional effects of changing sets of amino acid residues at the α7(+)/(−)β2 subunit interfaces (putative allosteric α‐CtxPnIC‐binding sites) to their non‐conserved equivalents at the α7(+)/(−)α7 interface (known orthosteric agonist‐binding site). (A) Concentration‐response curves (CRCs) for activation by the orthosteric agonist ACh were determined for each of the two α7β2‐nAChR variants and the unmodified α7β2‐nAChR construct. For each construct, agonist potency (EC_50_), maximum function (I_max_), and hillslope (n_H_) were determined by least‐squares curve‐fitting and are summarized in the Results section, along with the associated statistical analyses. Data points represent the mean ± SEM of 15–19 CRC measurements, each taken from an individual oocyte, across three separate batches of oocytes. (B) For the second‐most α7β2‐nAChR selective α‐CtxPnIC[S4R] analog (30 nM), kinetics of onset, and recovery from, antagonism were analyzed as described in the legends of Figures 3 and 4, providing values of the disassociation rate (*k*_*off*_), association rate (*k*_*on*_), and the disassociation constant (*K*_*d*_) at each α7β2‐nAChR construct (summarized in Table 3). (C) Values of *k*_*off*_, *k*_*on*_, and *K*_*d*_ were similarly determined for the most α7β2‐nAChR selective α‐CtxPnIC[L10Y] analog (100 nM), and are also summarized in Table 3. For both (B, C), statistical analyses are summarized in Table 3; data points are the mean ± SEM of 6–8 individual determinations, with each determination obtained from an individual oocyte, across three separate lots. All functional data were collected using two‐electrode voltage‐clamp electrophysiology.

First, we tested whether these changes altered concentration‐response relationships for ACh‐induced function compared to the unaltered α7β2‐nAChR. As illustrated in Figure 6A, the resulting concentration‐response curves (CRCs) are similar across the three constructs. Application of one‐way ANOVA to the maximum amount of function induced by ACh (I_max_), ACh EC_50_ values, or the hillslope of the CRCs (n_H_), indicated that none of these parameters differed significantly across the unaltered, outer pocket, and inner pocket α7β2‐nAChR constructs (respective values were: I_max_: 1353 ± 149 nA, 1401 ± 137 nA, 1539 ± 193 nA, F [2, 48] = 0.35, *p* = 0.71; EC_50_: 376 ± 43 μM, 365 ± 80 μM, 363 ± 46 μM, EC_50_: F [2, 48] = 0.012, *p* = .99; n_H_: 1.03 ± 0.07, 1.17 ± 0.12, 0.93 ± 0.06, F [2, 48] = 1.72, *p* = .19). These results indicate that the changes introduced at the α7(+)/(−)β2 subunit interfaces did not significantly affect agonist‐induced α7β2‐nAChR function.

The kinetics of antagonist onset and recovery were then compared between these two constructs, for the two α‐CtxPnIC analogs with greatest selectivity for α7β2‐ over α7‐only‐nAChR ([S4R] and [L10Y]). The resulting data are illustrated in Figure 6B,C, respectively, and the kinetic parameters arising from analysis of these outcomes are summarized in Table 3.

**TABLE 3:** Kinetic analyses of α‐CtxPnIC[S4R] and [L10Y] analog activity at unaltered or mutated α7β2‐nAChR constructs.

α‐CtxPnIC analogs		Unaltered α7β2‐nAChR	“Inner pocket” α7β2‐nAChR	“Outer pocket” α7β2‐nAChR
[S4R]	*k*_*off*_/min^−1^ (×10^−3^)	7.46 ± 0.98*	13.9 ± 2.7	15.3 ± 2.4
	*k*_*on*_/min^−1^ nM^−1^ (×10^−3^)	4.05 ± 0.65	2.73 ± 0.37	3.86 ± 0.99
	*K*_*d*_/nM	1.90 ± 0.21*	5.31 ± 1.04	6.74 ± 2.67
[L10Y]	*k*_*off*_/min^−1^ (×10^−3^)	30.5 ± 2.3	32.2 ± 6.4	38.4 ± 5.0
	*k*_*on*_/min^−1^ nM^−1^ (×10^−3^)	1.84 ± 0.33	1.30 ± 0.16	1.44 ± 0.19
	*K*_*d*_/nM	20 ± 4	24 ± 3	28 ± 4

*Note*: For the two the most α7β2‐selective α‐CtxPnIC analogs ([S4R] and [L10Y]), kinetics of recovery from, and onset of, antagonism were compared across three α7β2‐nAChR constructs. As outlined in the legend to Table 2, values of the true disassociation rate (*k*_*off*_), true association constant (*k*_*on*_), and disassociation constant (*K*_*d*_) were derived using multiple site kinetics models. Individual one‐way ANOVA analyses was used to assess whether each kinetic parameter, for each peptide analog, differed significantly across the three nAChR constructs. *Post hoc* analysis (Student‐Neuman‐Keuls pairwise comparison method) was applied in cases where the overall analysis indicated a statistically significant effect of construct. The [S4R] analog disassociated more slowly from the unaltered α7β2‐nAChR construct than from either of the binding pocket mutant constructs, resulting in lower affinity binding to the inner and outer pocket mutants compared to the unaltered construct (*k*_*off*_ and *K*_*d*_; *p < .05). No statistically‐significant effect of construct was observed on any kinetic parameter of α‐CtxPnIC[L10Y] antagonism. All data are the mean ± SEM of 6–8 individual determinations, corresponding to individual oocytes. F‐values, degrees of freedom, etc. for the individual ANOVA analyses are summarized in the Results section.

Considering first α‐CtxPnIC[S4R], the onset of antagonism appeared to follow a similar time course across the three versions of the α7β2‐nAChR construct. However, recovery from block seemed more rapid from both the inner pocket and outer pocket α7β2‐nAChR constructs than it was from the unaltered α7β2‐nAChR. Indeed, statistical analysis confirmed this: one‐way ANOVA showed a significant effect of construct on *k*_*off*_ (F [2, 18] = 4.74; *p* = .022). Further, *post hoc* analysis (Student–Newman–Keuls pairwise comparison method) indicated that *k*_*off*_ was statistically indistinguishable between the inner pocket and outer pocket α7β2‐nAChR constructs, and was faster in both cases than from the unaltered α7β2‐nAChR. When the resulting values of *k*_*obs*_ and *k*_*off*_ were used to calculate k_on_ (Table 3), one‐way ANOVA indicated that values of *k*_*on*_ for α‐CtxPnIC[S4R] were statistically indistinguishable across the three variants of the α7β2‐nAChR construct (F [2, 18] = 0.87; *p* = .436). Finally, one‐way ANOVA revealed a significant effect of construct on *K*_*d*_ (F [2, 18] = 4.62; *p* = .024). *Post hoc* analysis (Student–Newman–Keuls pairwise comparison method) indicated that the affinity of α‐CtxPnIC[S4R] binding at the unaltered α7β2‐nAChR was higher than at either the inner pocket or outer pocket variants, while *K*_*d*_ was indistinguishable between the inner pocket and outer pocket α7β2‐nAChR constructs. These results indicate that both the inner and outer pocket sets of amino acids at the β2(−) subunit face play significant roles in mediating α‐CtxPnIC[S4R] selectivity for α7β2‐ over α7‐only‐nAChR. In turn, this indicates that the α7β2‐nAChR selectivity of α‐CtxPnIC analogs arises from interactions with the α7(+)/(−)β2 subunit interfaces unique to α7β2‐nAChR. Reassuringly, in this set of experiments, the calculated *K*_*d*_ value of α‐CtxPnIC[S4R] binding to the unaltered α7β2‐nAChR construct (1.90 ± 0.21 nM) closely matched that calculated in the equivalent but separate set of experiments summarized in Table 2 (0.83 ± 0.05 nM), indicating the consistency of the kinetics analysis approach.

In a striking contrast, one‐way ANOVA showed no statistically‐significant differences of disassociation rate, association rate, or binding affinity of α‐CtxPnIC[L10Y] (*k*_*off*_, *k*_*on*_, or *K*_*d*_, respectively) across the three versions of the α7β2‐nAChR construct (*k*_*off*_, F [2, 19] = 1.44, *p* = .26; *k*_*on*_, F [2, 19] = 0.80, *p* = .45; *K*_*d*_, F [2, 19] = 0.83, *p* = .46). This finding, combined with the preceding results from the [S4R] analog, appears to indicate that other features of the α7(+)/(−)β2 subunit interfaces (i.e., not the inner and outer pocket sets of amino acid residues) are the major mediators of the α7β2‐nAChR selectivity of α‐CtxPnIC[L10Y]. The robustness of the kinetics analysis was again illustrated by the similar *K*_*d*_ values of α‐CtxPnIC[L10Y] binding to the unaltered α7β2‐nAChR construct, calculated in Table 2 (12 ± 3 nM) and in the separate set of experiments summarized in Table 3 (20 ± 4 nM).

### 3.6 SMD simulation reveals that [S4R] and [L10Y] substitutions affect the interactions of nAChR and α‐CtxPnIC analogs in different manners

**FIGURE 7:**
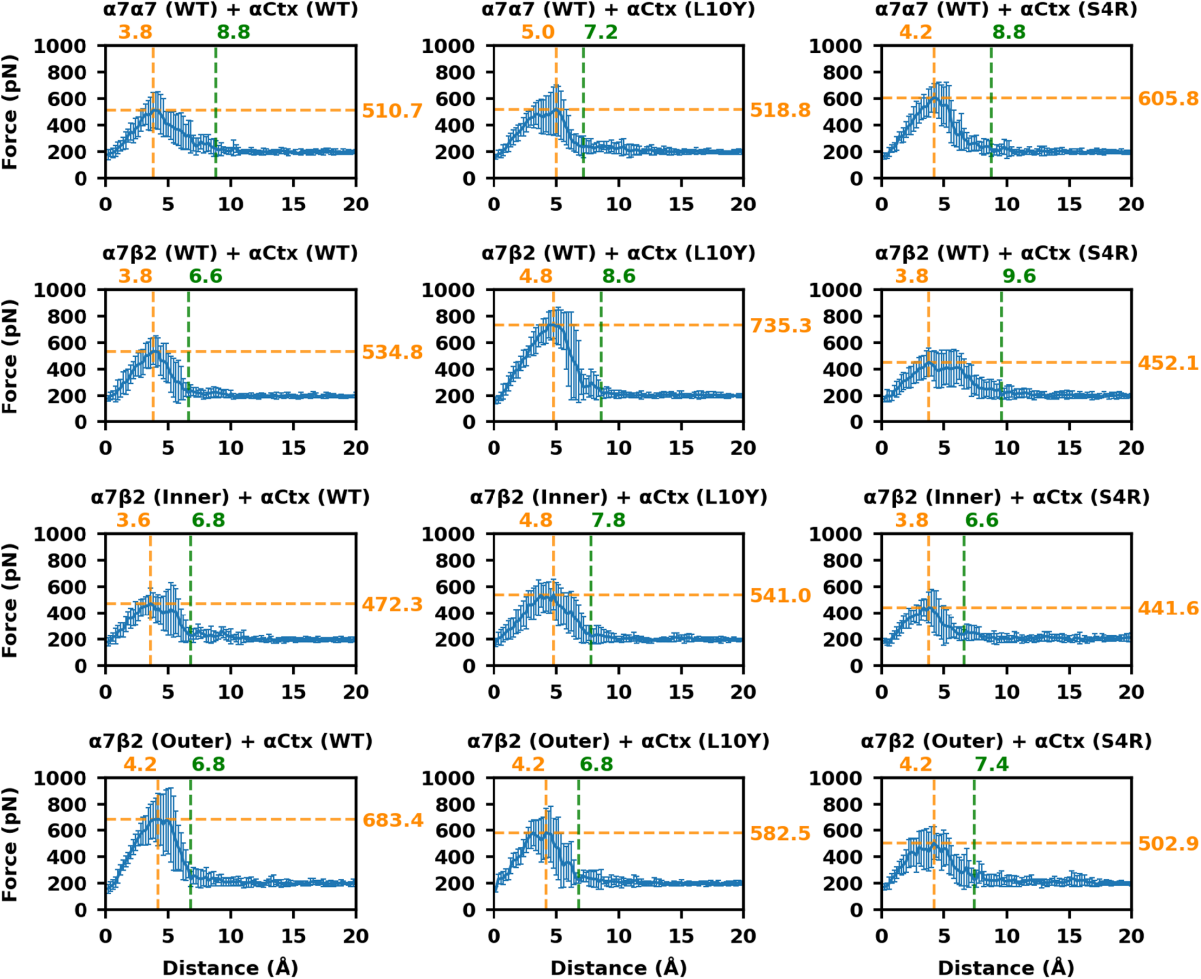
Force profiles of different combinations of nAChR and α‐CtxPnIC analogs as a function of the pulling distance: The *X*‐axis represents the displacement of α‐CtxPnIC COM with respect to the COM of nAChR pentamer. The *Y*‐axis represents the average and standard deviation of the effective pulling forces calculated from ten independent SMD simulations. The orange lines mark the peak of the pulling force, and the green lines mark the location where the pulling force drops to 10% away from the median.

To gain insight into potential differences between α‐CtxPnIC analogs in their interactions with α7(+)/(−)β2 versus α7(+)/(−)α7 nAChR‐binding interfaces, we performed SMD simulations in which we applied external forces to facilitate the dissociation of α‐CtxPnIC. We measured the magnitude of applied force as a function of α‐CtxPnIC displacement, and recorded the force peak location and the location where the force dropped to a steady state (Figure 7). The α7β2(WT)‐α‐CtxPnIC[L10Y] model system has the highest force peak, indicating that it requires the largest external force to dissociate α‐CtxPnIC[L10Y] from the α7(+)/(−)β2 subunit interface, and this may represent one of the reasons for the selectivity of α‐CtxPnIC[L10Y]. In the α7β2(WT)‐α‐CtxPnIC[S4R] system, the force dropped to the steady state when α‐CtxPnIC was displaced by 9.6 Å, which is a longer distance than that in the systems of α‐CtxPnIC[S4R] bound to α7*‐nAChR or α7β2‐nAChR with inner or outer mutations. This difference suggests that α‐CtxPnIC[S4R] maintains interaction with the α7(+)/(−)β2 subunit interface for a longer distance in the process of dissociation. These results indicate that [L10Y] and [S4R] both affect the binding interactions of nAChR and α‐CtxPnIC analogs, but that they may work through different mechanisms.

### 3.7 Off‐target effects of α‐CtxPnIC analogs on non‐α7*‐nAChR subtypes

Last, we determined whether α‐CtxPnIC analogs exhibited antagonist activity at other nAChR subtypes likely to be co‐expressed, and/or likely to exhibit overlapping pharmacology, with α7*‐nAChR. The subtypes investigated were α4β2‐nAChR (the most prevalent and widely distributed subtype expressed in the central nervous system^55^).

α3β2‐ and α6β2*‐nAChR (which are frequently sensitive to antagonism by α‐Ctx ligands with the same α4/7 fold as α‐CtxPnIC (i.e., peptide loop within first disulfide bridge = 4 amino acids in length, peptide loop within the second disulfide bridge = 7 amino acids^30^, ^39^, ^56^, ^57^, ^58^, ^59^).

α3β4‐nAChR (prominently expressed in the central and peripheral nervous systems^1^, ^55^).

α9α10‐nAChR (commonly co‐expressed with α7*‐nAChR in a wide range of non‐CNS locations, and exhibiting a similar pharmacological profile across a range of small ligands^60^, ^61^, ^62^, ^63^).

**FIGURE 8:**
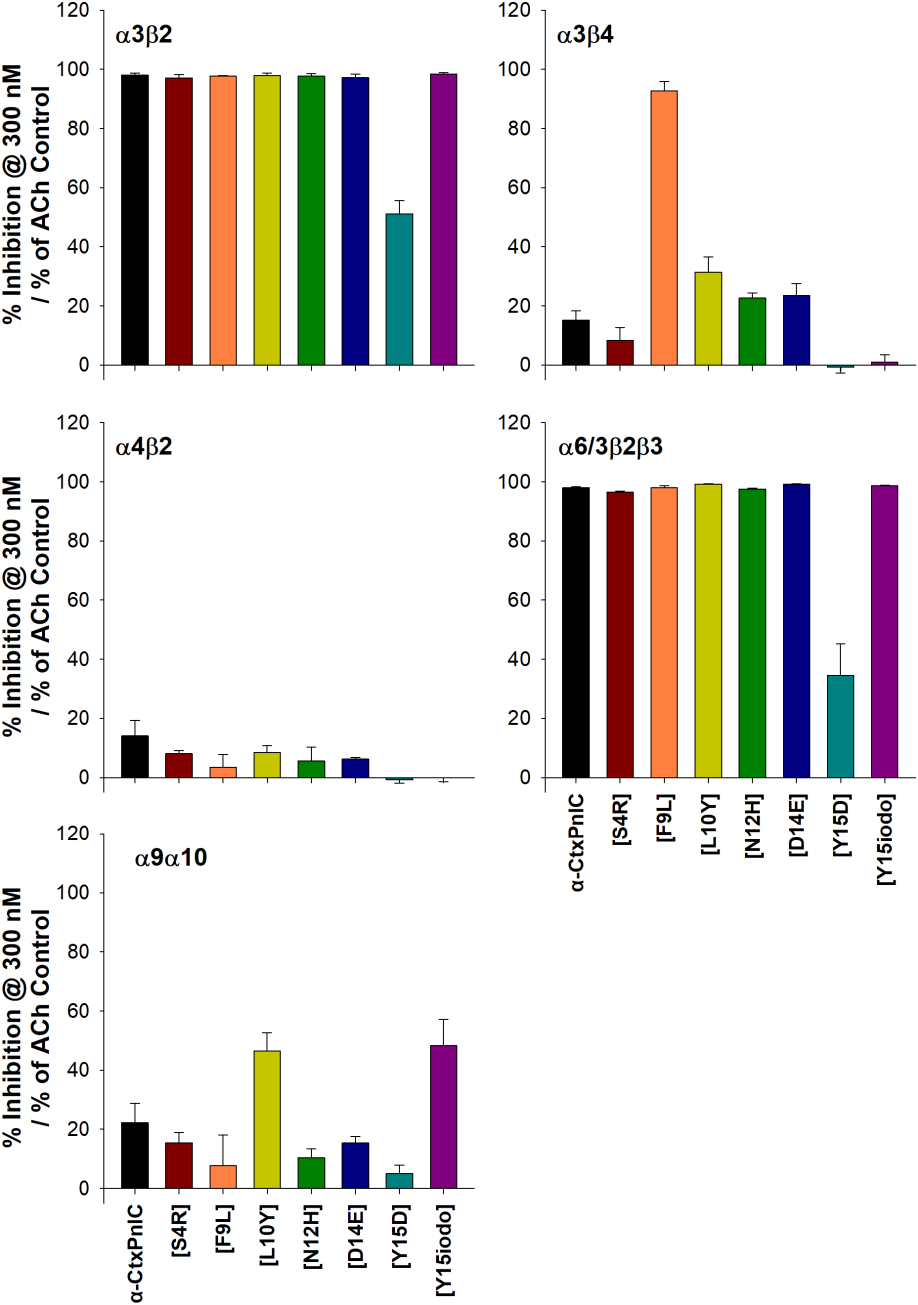
Antagonist efficacy of α‐CtxPnIC analogs at non‐α7*‐nAChR subtypes expressed in *X. laevis oocytes*: The ability of α‐CtxPnIC analogs (300 nM in all cases) to antagonize ACh‐induced function of non‐α7*‐nAChR subtypes (α3β2, α3β4, α4β2, α6/3β2β3, and α9α10) was assessed using two‐electrode voltage‐clamp electrophysiology. Bars represent the mean ± SEM inhibition of ACh‐induced function in each case, collected across four individual oocytes from different batches.

As detailed in the Methods section, antagonist activity of each α‐CtxPnIC analog was assessed across each of these subtypes, at 300 nM. The outcomes are illustrated in Figure 8. For the commonly‐expressed α3β4‐ and α4β2‐nAChR subtypes, even this very high concentration (≈100‐fold higher than the *K*_*d*_ values of most analogs at α7β2‐nAChR) produced ≤25% block of function, with only one exception (α‐CtxPnIC[F9L] induced >90% block at the α3β4‐nAChR subtype). Outcomes at α3β2‐ and α6/3β2β3‐nAChR were essentially the opposite: all but one of analogs produced >90% block at both subtypes (the exception being the [Y15D] analog, which exhibited ≈50% or ≈35% antagonist efficacy at α3β2‐nAChR or α6/3β2β3‐nAChR, respectively). Antagonist efficacy was modest at α9α10‐nAChR: most analogs produced 20% block or less of ACh‐induced function, with only the [L10Y] and [Y15iodo] analogs approaching 50% antagonist efficacy.

## 4 DISCUSSION

Employing a kinetics analysis approach provided significant mechanistic insights into how the selectivity of CtxPnIC analogs for α7β2‐ over α7‐only‐nAChR arises. For example, kinetics analysis showed that all α‐CtxPnIC analogs (with the exception of [F9L]) bound to approximately five sites on α7‐only‐nAChR (4.91 ± 0.38 sites; mean ± SEM), taking *N*_*obs*_ (number of sites bound to induce observed functional block) data from Table 2 and excluding *N*_*obs*_ data for the [F9L] analog), with occupation of multiple sites required to block function. This fits with the concept that all five α7/α7 subunit interfaces in the α7‐only‐nAChR subtype are available to bind agonist (or a competitive antagonist^39^). This requirement for multiple sites to be occupied by α‐CtxPnIC analogs in order to produce effective block of α7‐only‐nAChR was reinforced by a requirement for disassociation from only 2.15 ± 0.17 sites (again excluding data for the [F9L] analog); that is, α7‐only‐nAChR function can be restored even when multiple sites are occupied by an α‐CtxPnIC analog. In contrast, binding of the same analogs to only a single site was capable of effectively blocking function of α7β2‐nAChR, and onset of block was accelerated by the presence of what was calculated to be more than a single binding site (1.37 ± 0.06 sites). Restoration of α7β2‐nAChR function required disassociation from a similar number of sites (1.38 ± 0.09). Together, these observations explain the delayed onset of block at α7‐only‐compared to α7β2‐nAChR: block cannot commence at α7‐only‐nAChR until multiple (4‐to‐5) antagonist molecules have associated, whereas onset of α7β2‐nAChR block is initiated immediately by association of antagonist at any of 1‐to‐2 available sites. In a second example, the data summarized in Table 2 indicate that selectivity between α7‐only‐ and α7β2‐nAChR subtypes is primarily driven by faster disassociation of α‐CtxPnIC analogs from, and a corresponding loss of affinity at, α7‐only nAChR.

It is important to consider further the implications of these two different aspects of selectivity (binding affinity and kinetics) of α‐CtxPnIC analogs toward α7β2‐nAChR. Perhaps the simplest is differences in binding affinity (*K*_*d*_). Five α‐CtxPnIC analogs exhibited significantly higher binding affinity for α7β2‐ than α7‐only‐nAChR (the most‐selective analog [L10Y] exhibited nearly 60‐fold selectivity; Table 2). Even in this case, the presence of multiple antagonist‐binding sites per receptor has consequences for the relationship between the true disassociation constant (*K*_*d*_) and the observed antagonist potency (IC_50_). As outlined in our prior publication, apparent potency is enhanced in the presence of multiple binding sites.^39^ In addition, the impact of kinetic selectivity must also be considered. Due to the lag before antagonist activity begins at α7‐only‐nAChR, brief application of α‐CtxPnIC analogs can produce highly‐effective and long‐lasting block of α7β2‐nAChR, while having little effect on α7‐only‐nAChR function (see Figures 2 and 3). Indeed, this highlights an important point: it is likely that prolonged application of high concentrations of α‐CtxPnIC analogs to α7β2‐nAChR results in occupation of α7(+)/(−)α7 agonist‐binding sites. However, the combination of higher binding affinity, faster onset of functional block, and slower disassociation of α‐CtxPnIC analogs, at the α7(+)/(−)β2 non‐competitive binding site means that peptide action at α7(+)/(−)β2 sites dominates the functional effects of α‐CtxPnIC analogs at α7β2‐nAChR.

Multiple lines of evidence indicate that the non‐competitive binding site for α‐CtxPnIC analogs at α7β2‐nAChR is located at the α7(+)/(−)β2 subunit interfaces. The first is the indication of 1–2 sites being present on α7β2‐nAChR. This fits with the presence of two α7(+)/(−)β2 subunit interfaces in the α7β2‐nAChR constructs that we used. The second is the MD simulations, which indicate that α‐CtxPnIC itself and the two analogs with greatest selectivity toward α7β2‐nAChR ([S4R] and [L10Y]) all can form stable interactions at an α7(+)/(−)β2 subunit interface. The third and most compelling piece of evidence comes from the site‐directed mutation experiments that were informed by the MD simulations. It is important to note that neither of the sets of mutations (at the entrance to the putative α‐Ctx binding pocket at the α7(+)/(−)β2 subunit interface “outer pocket”), or deeper inside the interface (“inner pocket”) altered maximum function induced by ACh, nor its EC_50_ value, compared with the unmodified α7β2‐nAChR. This supports multiple earlier studies that indicate that agonist activation of α7β2‐nAChR is mediated only via sites at α7(+)/(−)α7 subunit interfaces,^16^, ^17^, ^18^ and reinforces the allosteric, non‐competitive nature of the α7(+)/(−)β2 interface binding site for α‐CtxPnIC analogs. However, both the inner and outer pocket sets of mutations significantly accelerated disassociation of the [S4R] analog from α7β2‐nAChR, without altering association kinetics. This constitutes direct evidence that α‐CxtPnIC analogs can bind selectively at the non‐agonist‐binding α7(+)/(−)β2 subunit interfaces of α7β2‐nAChR. As would be predicted for mutations that exchanged subsets of β2(−) residues for their α7(−) equivalents, the disassociation kinetics of the [S4R] analog from the inner and outer pocket mutant α7β2‐nAChR (see Table 3) were intermediate between those of unmodified α7β2‐nAChR (slower disassociation; see Table 2) and α7‐only‐nAChR (faster disassociation; see Table 2). Intriguingly, two earlier studies identified β2(−) residues V111 and F119 as contributors to kinetic differences of α‐CtxBuIA disassociation from β2*‐ versus β4*‐nAChR subtypes,^54^ and as contributors to agonist affinity differences between α3β4‐ and α4β2‐nAChR.^64^ In the current study, β2(−) residues V111 and F119 are also among those that are mutated (in this case to their α7(−) equivalents) in the inner and outer pocket sets of mutations. The engagement of the same number of sites of action on α7β2‐nAChR by α‐CtxPnIC[L10Y] as the [S4R] analog (see Table 2) suggests that it, too, generates selectivity by interacting at the α7(+)/(−)β2 subunit interfaces. However, the lack of effect of the inner and outer pocket mutations on kinetics of α‐CtxPnIC[L10Y] association or disassociation rates suggests that different interactions at the α7(+)/(−)β2 interface may drive α7β2‐nAChR selectivity for this analog. This perspective is reinforced by the SMD outcomes indicating that the α‐CtxPnIC[S4R] and [L10Y] analogs may generate selectivity toward α7β2‐nAChR through different mechanisms (see Figure 7). Since the α‐CtxPnIC fourth and tenth amino acid positions are located at opposite ends of the folded peptide (see Figures 5 and S1), these conclusions are perhaps unsurprising. Although the SMD simulations provided some potentially valuable insights, there are some caveats to consider. For example, the force profiles from SMD simulations can only partially explain the differences in the disassociation rate (*k*_*off*_) measured from experiments. Additionally, in the SMD simulations, we only calculated the external force required to dissociate one α‐CtxPnIC analog from one binding interface, while α7‐only‐ and α7β2‐nAChR have five lower affinity or two higher affinity peptide‐binding interfaces, respectively.

Extensive prior experience^33^, ^39^, ^65^, ^66^, ^67^ indicates that α‐Ctx peptides typically interact with a nAChR subtype of interest with high affinity, and may also have significant affinity at one or two additional nAChR subtypes. The family of α‐CtxPnIC analogs described in the current study conforms to this typical pattern. As illustrated in Figure 8, application of analogs at even 300 nM (far above the effective concentrations of these peptides at the intended α7β2‐nAChR target) generally had relatively little effect on agonist‐induced function of α3β4‐, α4β2‐, or α9α10‐nAChR. The few exceptions were a maximum ≤50% block by 300 nM [L10Y] and [Y15iodo] analogs at α9α10‐nAChR (which indicates IC_50_ values >300 nM at this subtype versus ≈1–2 nM or ≈10 nM affinity at α7β2‐nAChR, respectively for these analogs), and good‐efficacy block of α3β4‐nAChR function by the [F9L] analog (which is of low interest since it also is not selective between α7‐only‐ and α7β2‐nAChR). However, potency at α3β2‐ and α6/3β2β3‐nAChR subtypes was generally high across the set of α‐CtxPnIC analogs. Even now, the ≈60‐fold selectivity of α‐CtxPnIC[L10Y] for α7β2‐ over α7‐nAChR is likely sufficient to be of practical use in regions like the basal forebrain cholinergic nuclei, where α4β2‐nAChR is the only other subtype commonly expressed.^21^ Fortunately, it is possible to enhance desired subtype affinities and engineer out those that are not wanted. This approach routinely has produced ≥1000‐fold selectivity toward intended nAChR subtypes over all others^33^, ^39^, ^65^, ^66^, ^67^; effectively complete specificity in real‐world use. Indeed, the data collected here indicate a clear path forward to develop far‐more α7β2‐nAChR‐selective analogs of α‐CtxPnIC. Combining amino acid substitutions that individually produce α7β2‐nAChR preference will greatly increase selectivity for α7β2‐ over α7‐only‐nAChR, since selectivity conferred by individual analogs typically multiplies together, rather than simply adds (see preceding citations). Our data indicate that α7β2‐ over α7‐only‐nAChR selectivity is mediated by different peptide‐receptor interactions for the two most‐discriminating analogs identified so far ([S4R] and [L10Y]). This finding suggests that a combinatorial approach has the opportunity to be as successful for α‐CtxPnIC as it has been for other α‐Ctxs that have been developed previously. In addition, the [Y15D] substitution reduces potency at all non‐α7*‐nAChR subtypes substantially below their potency at α7β2‐nAChR, while also providing an additional minor degree of preference for α7β2‐ over α7‐only‐nAChR. It is important to note that these highly‐promising insights have been obtained from the initial set of eight lead peptides identified in our functional screen. More extensive structure–function studies will reveal many further opportunities to enhance selectivity of α‐CtxPnIC analogs toward α7β2‐nAChR. The resulting data will also provide extensive experimental constraints on future MD simulations, providing much more refined insights into the precise nature of the interactions that enhance antagonist selectivity and efficacy of α‐CtxPnIC analogs at α7(+)/(−)β2 over α7(+)/(−)β2 nAChR subunit interfaces.

In conclusion, we have discovered the first family of α4/7 α‐Ctx analogs with selectivity toward α7β2‐ over α7‐only‐nAChR, and demonstrated that they already show promising selectivity away from other non‐α7‐nAChR subtypes. We have also demonstrated that, unlike previously characterized α‐Ctx ligands which act as competitive antagonists,^5^ the α7β2‐nAChR selectivity of α‐CtxPnIC analogs arises from a novel non‐competitive mechanism. This discovery opens up two highly significant new areas of research. The discovery of novel α7β2‐nAChR selective ligands opens, for the first time, a route toward interrogating the specific roles of α7‐only‐ versus α7β2‐nAChR subtypes. Given the links between oAβ_42_/α7β2‐nAChR interactions on basal forebrain cholinergic neurons to critical events in early AD, and the more restricted distribution of α7β2‐ versus α7‐only‐nAChR,^17^, ^19^, ^20^, ^21^, ^23^ α7β2‐nAChR could be an exceptionally significant pharmacotherapeutic target. In addition, neither nAChR harboring accessory subunits, nor most nAChR isoforms composed of different stoichiometries of the same subunits, can be positively identified and therefore easily studied in native systems. This study provides a prototypical illustration of where, and through which interactions with a nAChR target, α‐Ctxs can be targeted to noncanonical interfaces, thereby discriminating between related nAChR complexes that otherwise present identical competitive agonist‐binding interfaces. This finding therefore fundamentally expands the scope of potential α‐Ctx applications, with the promise to enhance significantly the already profound impact of α‐Ctx peptides on nAChR research and translational applications.

## AUTHOR CONTRIBUTIONS

Paul Whiteaker, Wonpil Im, Baldomero M. Olivera, and J. Michael McIntosh conceived and designed the research with significant assistance from Andrew A. George and Linda M. Lucero regarding design of the kinetics experiments; Andrew A. George, Sabin J. John, Linda M. Lucero, J. Brek Eaton, Ekta Jaiswal, Sean B. Christensen, Joanna Gajewiak, Maren Watkins, and Yiwei Cao performed the research and acquired the data; all authors were involved in analyzing and interpreting the data; all authors were involved preparing and revising the manuscript.

## DISCLOSURES

The authors declare no conflicts of interest.

## Supporting information


Supplementary Figure S1.

## Data Availability

The data that support the findings of this study are available in the methods and/or supplementary material of this article.
