# Supplementary material for: Analogs of α‐conotoxin PnIC selectively inhibit α7β2‐ over α7‐only subtype nicotinic acetylcholine receptors via a novel allosteric mechanism
Source: FASEB J. 2023 Dec 31;38(1):e23374. doi: 10.1096/fj.202302079 (PMC10782225; doi:10.1096/fj.202302079)
Supplement: Supplementary file 1 — Supplementary Figure S1. [file FSB2-38-e23374-s001.pdf]

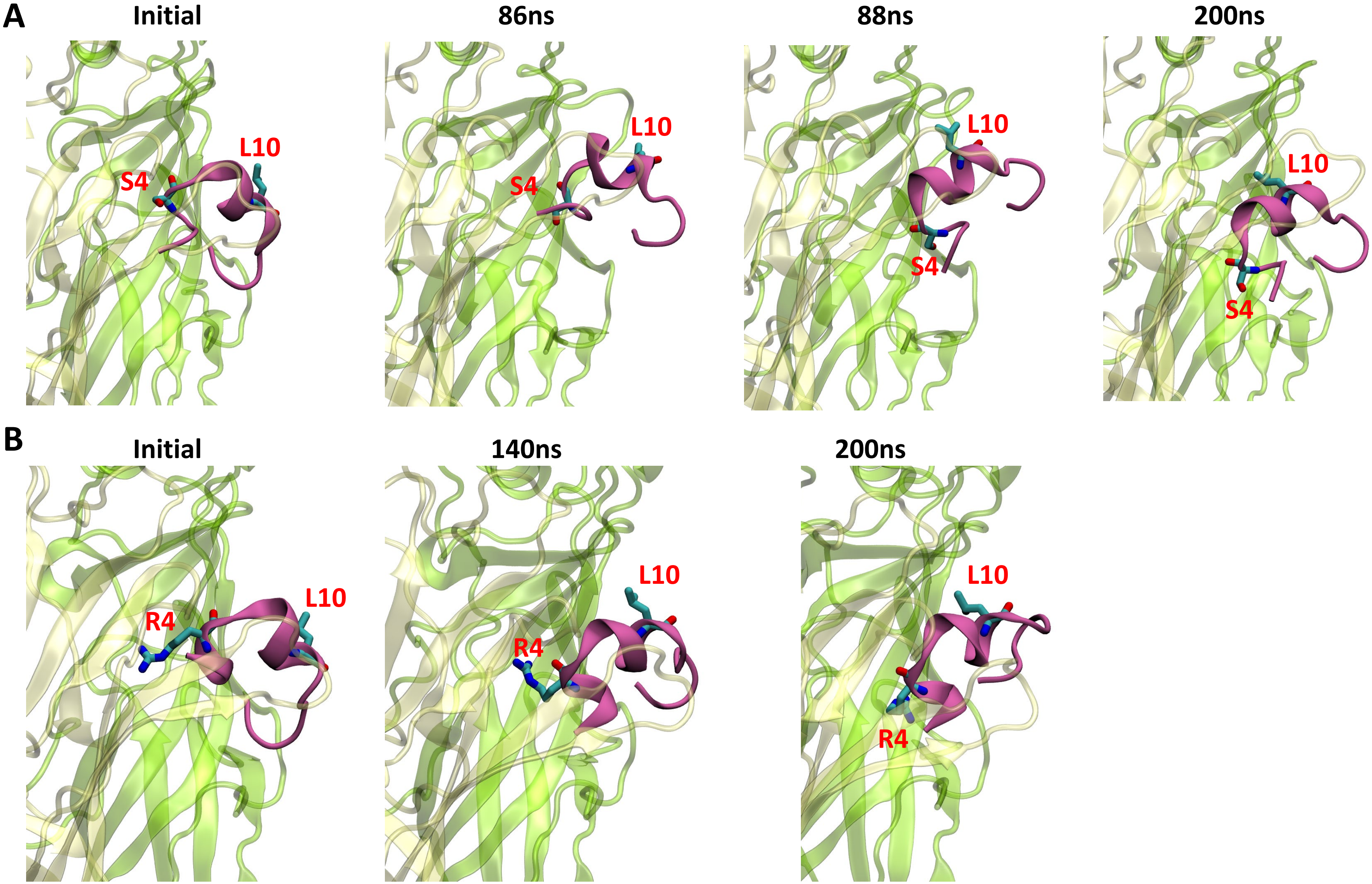

Supplementary Figure S1. Representative snapshots from simulations starting from nAChR in complex with α-CtxPnIC in an upside-down binding pose. A) α-CtxPnIC bound to the α7(+)/(-)β2 interface. B) α-CtxPnIC[S4R] bound to the α7(+)/(-)β2 interface. The snapshots are extracted from simulations of 200 ns. α-CtxPnIC (WT and S4R) started from the upside-down binding pose and flipped rapidly back to the common binding pose overserved in the PDB structures of nAChR/AChBP-α-conotoxin complexes.

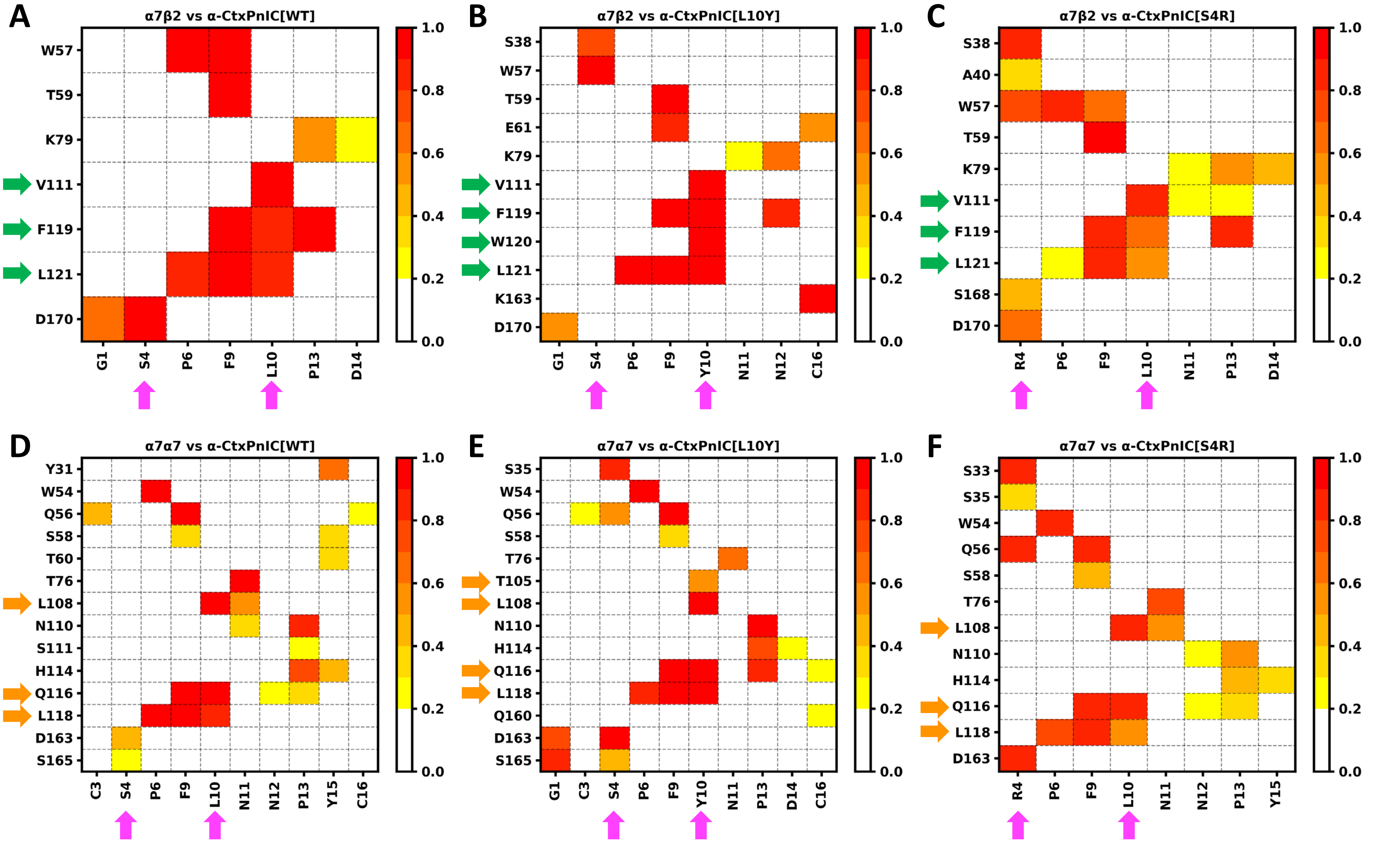

Supplementary Figure S2. The contact maps of residues in nAChR-α-CtxPnIC binding interfaces. A-C) α-CtxPnIC (WT, L10Y and S4R) bound to the α7(+)/(-)β2 interface. D-F) α-CtxPnIC (WT, L10Y and S4R) bound to the α7(+)/(-)α7 interface. The X-axis shows the residues of α-CtxPnIC analogs, and the Y-axis shows the residues of α7(-) subunit at α7(+)/(-)α7 interface, or the residues of β2(-) subunit at the α7(+)/(-)β2 interface respectively. Only the residue pairs making side-chain interactions in at least 20% of the simulation time are shown here, and the color bar represents the frequency of interaction throughout the simulation period (1 μs).
